# Emotional Regulation in Substance-Related and Addictive Disorders Treatment: A Systematic Review

**DOI:** 10.1007/s10899-024-10366-8

**Published:** 2025-02-07

**Authors:** Samuel Chrétien, Isabelle Giroux, Isabelle Smith, Christian Jacques, Francine Ferland, Serge Sévigny, Stéphane Bouchard

**Affiliations:** 1 GRIF-Jeu, CQEPTJ, École de psychologie, Faculté des sciences sociales, Université Laval, Pavillon Félix-Antoine-Savard, Bureau 1336, 2325, Allée des Bibliothèques, Québec, QC G1V 0A6 Canada; 2 GRIF-Jeu, HERMES, Service de recherche en dépendance CIUSSS de la Capitale-Nationale/CISSS de Chaudière-Appalaches, Centre de recherche du CISSS-CA, Centre de services de réadaptation en dépendance du CIUSSS de la Capitale-Nationale, Québec, Canada; 3 GRIF-Jeu, CQEPTJ, Faculté des sciences de l’éducation, Département des fondements et pratiques en éducation, Université Laval, Québec, Canada; 4 GRIF-Jeu, Département de psychoéducation et de psychologie, Université du Québec en Outaouais, Québec, Canada

**Keywords:** Emotional regulation, Systematic review, Intervention, Non-substance-related disorders, Substance use disorders, Addictive disorders

## Abstract

Emotions play an undeniable role in addictive disorders. Given the high relapse and drop-out rates still prevalent in current treatments, it is crucial to explore curative alternatives that take greater account of emotions. The primary objective of this systematic review is to gather literature related to emotion regulation in psychological addictive disorders treatments. The aim is to describe its use for individuals with behavioral (such as gambling disorder, problematic Internet gaming, and Internet addiction) or substance-related disorders. Following a screening of nearly 12,000 articles from six databases and the grey literature, 38 studies that met the selection criteria were included. The results show that 63.2% of the studies had a psychological treatment predominantly based on emotional regulation, with 81.6% (*n* = 31) of third-wave cognitive-behavioral interventions. The most frequently utilized intervention techniques for emotional regulation were those that facilitated an individual's awareness of their emotional state or provided psychological education to assist in identifying emotions. It would be valuable for future research to explore the most effective content for emotional regulation in treating substance-related and addictive disorders and to determine the specific client population that would benefit the most from this treatment.

**Supplementary Information:**

The online version contains supplementary material available at 10.1007/s10899-024-10366-8.

## Introduction

Substance use disorders (SUD) and behavioral addictive disorders have various consequences that affect physical and psychological health (Kessler, 2004; NIDA, 2011), interpersonal relationships (Mueller et al., 2009), work (Baldwin & Marcus, 2014) or finances (Fong, 2005). Substance use disorders (SUD) are characterized by the ongoing use of a psychoactive substance (PAS; alcohol, cannabis, etc.), as indicated on the DSM-5 by the presence of cognitive, behavioral, and physiological symptoms, despite significant consequences (APA, 2013). Statistics Canada (2015) estimates the lifetime prevalence of SUD among people aged 15 and over at 21.6%.

Behavioral addictive disorders refer to a compulsive, dysfunctional engagement in repetitive, maladaptive actions, initiated by an uncontrollable impulse, to reduce discomfort or create a feeling of euphoria without involving the consumption of PAS (Karim & Chaudhri, 2012; Yau & Potenza, 2015). Behavioral addictive disorders include, among others, gambling disorder (GD; APA, 2013), excessive Internet use or Internet addiction (IA; Chamberlain et al., 2016), and Internet gaming disorder (IGD; APA, 2013). The prevalence of GD has been estimated at 1.29% of the adult population, according to a recent meta-analysis (Gabellini et al., 2023). The prevalence of IGD has been evaluated to be between 0.3 and 1.0% (Przybylski et al., 2017), and the prevalence of IA has been estimated to be between 1.5 and 8.2% (Weinstein & Lejoyeux, 2010).

Cognitive behavioral therapy (CBT) is a widely used approach for treating substance use disorders (SUD) and behavioral addictions (Cowlishaw et al., 2012; Yeterian et al., 2016; Young, 2013). CBT is a well-established group of psychological treatments that has developed through multiple generations, known as waves (Hayes & Hoffmann, 2017). The first wave, behavior therapy, aimed to modify observable behavior using learning principles. The second wave, classic CBT, highlights how maladaptive thinking patterns influence emotions and behavior, along with methods to modify these patterns (Hayes & Hofmann, 2017). A key component of CBT, particularly for GD, is modifying erroneous thoughts or false beliefs through cognitive restructuring (Fortune & Goodie, 2012; Ladouceur et al., 1998). CBT includes several techniques, such as psychoeducation, analyzing risk situations and triggers, training in problem-solving and social skills, exposure to situations likely to trigger cravings, and learning coping strategies (Gooding & Tarrier, 2009). CBT is effective in the short term (up to six months) for reducing symptoms and behaviors associated with addictive disorders (Cowlishaw et al., 2012; Yeterian et al., 2016; Young, 2013). However, its long-term efficacy remains uncertain.

While CBT remains an effective treatment for addictive disorders (Cowlishaw et al., 2012; Yeterian et al., 2016; Young, 2013), there is a notably high dropout rate associated with treatment for SUD and behavioral addiction (23–50% for outpatient SUD; Brorson et al., 2013; 31% for GD; Melville et al., 2007). Additionally, the presence of significant comorbidities may suggest poorer treatment outcomes (Chen et al., 2011; Starcevic & Khazaal, 2017). For example, Grant and Chamberlain (2015) found that among individuals seeking treatment, the rate of comorbidity between GD and SUD ranges from 35 to 63%. Furthermore, Starcevic and Khazaal (2017) reported that IGD is associated with higher levels of depression, anxiety, social anxiety, and symptoms of inattention and ADHD. The co-occurrence of personality disorders and SUD is common, with rates ranging from 50 to 92%, depending on the type of personality disorder, the studied population, and the sampling methods (Stetsiv et al., 2023).

These characteristics associated with SUD and behavioral addictions justify the exploration of complementary treatment approaches. Research shows a connection between how people regulate their emotions and their use of substances (Weiss et al., 2022). Leahy (2012) suggests that psychological disorders, such as SUD, can be understood as ineffective strategies for regulating emotions. These strategies may include rumination, worry, binge eating, suppression, and various forms of experiential avoidance, all of which stem from dysfunctional responses to emotional experiences. There is a growing consensus that emotions serve a functional and regulatory role and are inherently interconnected with other processes, such as cognition. This has led to a shift in treatment approaches to incorporate this understanding (Mennin & Farach, 2007). McRae and Gross (2020) define ER as an individual's attempt to modify the polarized valence of emotions experienced during a situation. Emotions play an active role in the development and perpetuation of addictive disorders (Cheetham et al., 2010; Wong et al., 2018). In users of PAS, Lillaz and Varescon (2012) observed that a lack of emotional introspection (i.e., awareness, identification, and expression of emotions) seems to increase vulnerability to their use. Negative psychological states also play a role in the severity of addictive disorders. Wong et al. (2018) found that, among a sample of 177 persons with GD from Hong Kong seeking professional help, emotions help moderate the relationship between gambling-related cognitions and the severity of GD. Specifically, participants who reported higher levels of stress experienced more persistent and severe GD compared to those with lower stress levels, regardless of their gambling-related thoughts. In their systematic review, Marchica et al. (2019) demonstrated that individuals with low ER tend to exhibit more symptoms of GD and IGD. Additionally, the meta-analysis conducted by Stellern et al. (2023) observed that individuals with SUD appear to face greater challenges in regulating their emotions compared to those without SUD.

Focusing on emotions in substance-related and addictive disorders intervention is a sensible approach. Clinicians have begun incorporating third-wave CBT in their treatment protocols to primarily focus on ER (Dumont et al., 2016). The third wave of CBT emphasizes the relationship with thoughts and emotions rather than their content (Hayes, 2004) and has demonstrated positive effects. These mindfulness-based approaches encompass a diverse range of therapies, including Dialectical Behavior Therapy (DBT), Mindfulness-Based Cognitive Therapy (MBCT), and Acceptance and Commitment Therapy (ACT; Brown et al., 2011). These therapies highlight the significance of emotions in understanding psychopathology (Dimeff et al., 2021; Rector, 2010) and can serve as a complementary approach to second-wave therapies, which concentrate more on individual thoughts (Dionne et al., 2010). Intervention techniques include accepting one's emotions without judgment, being fully aware of them, and identifying, recognizing, and expressing emotional states (Dionne et al., 2010). Learning to manage emotions has also been shown to improve the effectiveness of CBT (Berking et al., 2008). In their study of 289 patients hospitalized for a mental health disorder, Berking et al. (2008) found that learning to manage emotions effectively reduced depression and increased positive affect. Systematic reviews of mindfulness interventions to treat SUD and GD have shown improved ER and reduced symptoms (Li et al., 2017; Sancho et al., 2018). Interventions aim to temporarily halt dwelling on the past, planning, and avoiding negative emotions such as pain, craving, and sadness (Korecki et al., 2020). Third-wave interventions are widely utilized in clinics, schools, communities, and online (Perkins et al., 2023).

Systematic reviews on emotions and addictive disorders focus solely on mindfulness interventions, particularly for SUD, while excluding those where ER is only a part of the treatment. Enhanced CBT, for example, is one of these treatments. It focuses on increasing ER skills, particularly for eating disorders (Fairburn et al., 2009). Classifying treatments according to their emphasis on ER would enable us to report their effectiveness. Similar to the approach taken by Korecki et al. (2020), who examined the treatment protocols of manualized mindfulness interventions for SUD on a session-by-session basis, describing the content of ER interventions for people with SUD or addictive disorders would allow for a critical assessment.

### Objectives

The review aims to gather literature related to emotion regulation in psychological substance-related and addictive disorders treatments. The objective is to describe its use for individuals with substance-related (all PAS included) or behavioral addictive disorders (GD, IGD, and IA). It also aims to describe the intervention content and report on effectiveness.

## Method

### Search Strategy

Between January 31 and August 15, 2022, we conducted searches for documents in Medline (Ovid), PsycINFO (Ovid), Web of Science (Clarivate), Psychology and Behavioral Sciences Collection (EBSCO), Embase (Elsevier), and Dissertations & Theses Global (ProQuest). We also searched for grey literature on Google Scholar, Santécom, OpenGrey, ClinicalTrial.gov, electronic theses and dissertations, and solicited North American addictive disorders research and treatment centers via email. The references cited in the selected documents have been reviewed. Three categories of keywords guided the selection of equations: Emotional regulation, addictions, and treatments (see “Appendix” for full search strategy). The method is based on the criteria of The Cochrane Collaboration (2008) and follows the presentation recommendations of *Preferred Reporting Items for Systematic Reviews and Meta-Analysis* (PRISMA; Page et al., 2021). The protocol was registered on PROSPERO (CRD42022362766).

### Eligibility Criteria

The inclusion criteria for the articles were to (a) be written in French or English; (b) use ER in psychological addictive disorders treatment; (c) include participants with behavioral addictive disorders (GD, IGD, or IA) or SUD, without age or ethnicity restrictions; (d) be written between the years 2000 and 2022; (e) report on a treatment, intervention or program and not just secondary analyses; and (f) had results of a post-treatment assessment of SUD or addictive disorders. Articles related to (a) pharmacological treatments and (b) systematic reviews or meta-analyses were excluded.

### Study Screening and Extraction

**Fig. 1:**
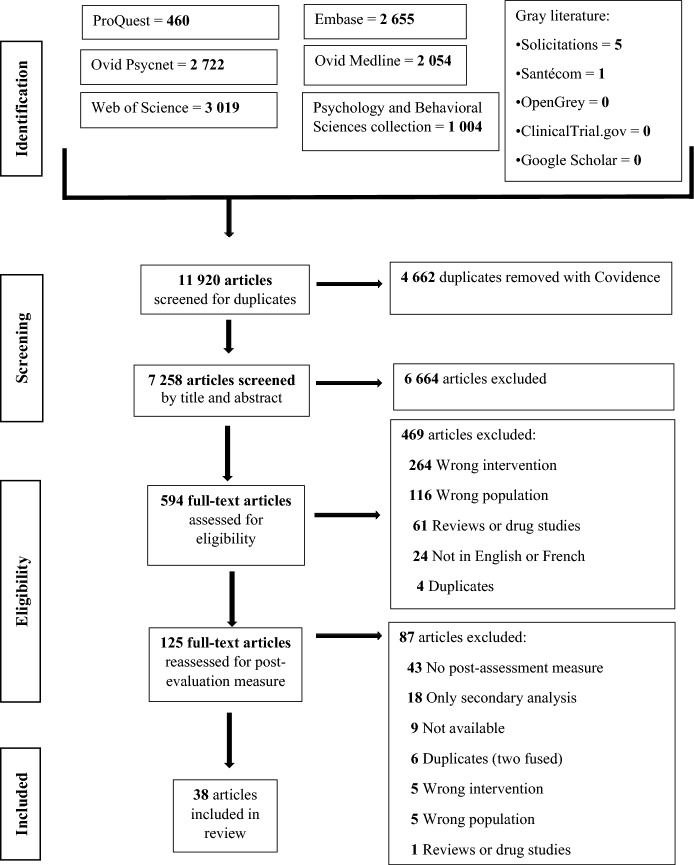
PRISMA flow diagram of study selection

The studies underwent a four-stage selection process, which included the identification of documents, selection, categorization, and final inclusion (Gedda, 2015). Covidence (Veritas Health Innovation, 2021) facilitated the sorting, grouping, and recording of titles, abstracts, and entire texts, as well as the removal of duplicates. Documents were screened for eligibility in three stages: (a) titles and abstracts were read; (b) selected documents were read in their entirety; and (c) a third screening was carried out, requiring a post-evaluation measure of symptomology (see Fig. 1). Two independent reviewers (psychology students and one author, IG) screened each study, and a third reviewer (the first author, SC) resolved disagreements. Data extraction was conducted in Covidence for types of treatment/intervention and objectives, techniques and tools used to regulate emotions, intervention modalities, duration, frequency and intensity, instruments, and effect of interventions on ER and symptomology.

### Categorization According to ER Intervention Content

After the extraction was completed, studies were found to vary according to their intervention content: Some treatments focused entirely on ER, while others addressed ER only briefly. Consequently, the studies were categorized based on the content of the intervention and the percentage of ER sessions. They were grouped into three categories: (a) Predominantly ER (more than 50% of sessions); (b) Partially ER (equivalent or less than another component) or (c) Contribution to ER not measurable, when each ER session’s content was not available.

### Quality Assessment

The risk of bias was assessed using a tool inspired by *The Cochrane Collaboration's tool for assessing risk of bias* (The Cochrane Collaboration, 2008) for randomized controlled studies, and the *Quality Assessment Tool for Quantitative Studies* (Effective Public Health Practice Project [EPHPP], 2021) for other specifications. The assessment underwent inter-rater agreement (verified by SC) until a consensus was reached.

## Results

### Study Characteristics

After reviewing 11,920 documents, 38 studies were included in the final sample (see Fig. 1). The studies are presented in Tables 1 and 2 and numbered in the reference list. Of the 38 studies, 78.9% focused on SUD, specifically alcohol (*n* = 13 ^4,10,11,14,15,16,19,26,27,30,31,32,35^), opioids (*n* = 4^3,12,17,36^), cannabis (*n* = 1^34^), nicotine (*n* = 3^7,8,18^), ketamine (*n* = 1^25^) or different substances simultaneously (*n* = 8^2,5,9,13,22,23,33,37^). The remaining studies (21.1%) addressed behavioral addictive disorders, encompassing GD (*n* = 5^6,20,24,29,38^), IA and IGD without distinguishing between them (*n* = 3^1,21,28^). In 78.9% of the studies, clinical interviews were conducted to establish a diagnosis using DSM criteria or a validated instrument (see Table 1). Study samples included 1–244 participants, aged 9–75. One study included children and adolescents exclusively^28^. Women comprised 41.0% of the total participants. Almost half of the studies (44.7%, *n* = 17) reported at least one comorbidity, such as a mood disorder, anxiety disorder, or borderline personality disorder (see Table 1).

**Table 1:** Countries, substance-related or non-substance-related disorders, study objectives, research design, inclusion and exclusion criteria, sample characteristics, ER and symptomology measures, and main results per study

Source ^#^Author(s) (year)	Country	SUD or NSRD		Study objectives	Research design	Criteria		Sample Intervention *N* Age in years: $\overline{X }$ *(SD* or *R*) *(SD* or *R*) Gender (% women) Dropout rate (in %)		ER And Symptomology Measures Instruments (time points)	Main results **Beneficial** *Neutral/no effect* ***Harmful***
		Diagnostic measures	Comorbidities			Inclusion	Exclusion	Exp	Con		
^1^Amini et al. (2020)	Iran	IUD (NR)	NR	Evaluate the effectiveness of EFGT in:	Controlled clinical trial	University student	Refusal to participate	EFGT	No intervention	IAT, PANAS (Pre-test, 13 weeks)	**↓ Negative emotions**
				↓Negative emotions		18 years or older		*n* = 20	*n* = 20		**↓ IUD symptoms**
				↓IUD symptoms		No severe psychiatric disorders		22.2 years (NR)	22.0 years (NR)		**↑ Positive emotions**
				↑Emotional excitement		Show symptoms of IUD		NR	NR		
								DR = 20.0	DR = 0.0		
^2^Axelrod et al. (2011)	USA	SUD-Alcohol, Cocaine, Opioids, Cannabis (DSM-IV)	BPD (100%) and Depression, Anxiety, PTSD, BD	Assess if DBT:	Cohort (pre/post)	Woman	NR	DBT	n/a	DERS, Substance use frequency, Urine toxicology screening, Alcohol testing (Pre-test, 10 weeks, 20 weeks)	**↑ ER**
				↑ER		Admitted to a primary substance use clinic		*n* = 27			**↑ Mood**
				Examine the relationship between ↑ER and SUD		Having BPD and SUD according to DSM-IV		38.0 years (NR)			**↓ Frequency of alcohol and drug use**
								100			
								DR = 44.4			
^3^Azizi et al. (2010)	Iran	SUD-Opioids (DSM-IV)	No	Investigate the effectiveness of and DBT-ER training therapy against standard behavioral group therapy on:	Controlled clinical trial	SUD according to SCID-I	Severe mental illness	#1: DBT mindfulness, ER skills training and naltrexone	Naltrexone	*The Opiate Treatment Index*, *Distress Tolerance Scale*; DERS (First and tenth sessions, frequency of sessions not specified)	**Experimental condition #1 > Experimental condition #2 > Control condition:**
				↑ ER enhancement		20–45 years old	Actively suicidal	*n* = 13	*n* = 13		**↑ Distress tolerance**
				↑ Distress tolerance		No comorbidities with certain psychiatric disorders	Not speaking English	25.6 years (NR)	27.7 years (NR)		**↑ ER**
				Relapse prevention		Not actively involved in psychotherapy		0	0		**Experimental conditions > Control condition:**
								DR = 0.0	DR = 7.7		**↓ Drug abuse**
											**↑ Health**
											**↑ Social functioning**
								#2: Beck cognitive group therapy and naltrexone			**↓ Depression**
								*n* = 13			
								26.7 years (NR)			
								0			
								DR = 0.0			
^4^Cavicchioli et al. (2019)	Italy	SUD-Alcohol (DSM-5)	SUD, GD, Mood, Anxiety, Eating and, Personality Disorders	In people with a primary diagnosis of SUD-alcohol during an outpatient treatment of DBT-ST, investigate changes in:	Cohort (pre/post)	DSM-5 for alcohol use disorder	Psychotic disorder or severe cognitive impairment	DBT-ST	n/a	Urine toxicology screening, SPQ-alcohol, SPQ-drugs, SPQ-drugs, DERS (Pre-test, 9 weeks)	**↑ Continuous days of abstinence**
				Consecutive days of abstinence			Active psychiatric comorbidity	*n* = 108			**↓ SUD-Alcohol**
				Severity of SUD				48.43 years (9.61)			**↓ Concomitant SUD**
				Difficulties in ER				40.7			**↑ ER**
				Use of maladaptive coping strategies				DR = 4.7			**↑ Use of DBT skills**
^5^Choopan et al. (2016)	Iran	SUD-Opioids (DSM-IV-TR)	NR	Determine if the ER training based on Gross's model:	Controlled clinical trial	Man	NR	ER training based on the Gross model	NR	Cravings beliefs questionnaire (Pre-test, NR)	**↓ Craving beliefs**
				↓Craving beliefs		18–50 years old		*n* = 15	*n* = 15		
						Drug dependence (DSM-IV-TR)		32.33 years (6.62)	NR		
						No severe psychological disorders		0	0		
						No physical illnesses that would preclude participation		DR = NR	DR = NR		
^6^Christensen et al. (2013)	Australia	Problem gambling (NR)	Suicidal ideation, BPD, High distress	Investigate the effectiveness of brief DBT for PG on the four DBT skills:	Cohort (pre/post)	Being a *Gambler's Help* Service customer and having made little progress	NR	Brief DBT	n/a	Gambling sessions, Amount spent DERS-ED (Pre-test, 9 weeks)	**↑ Abstinence**
				Mindfulness		Significant issues with ER and impulse control		*n* = 29			**↑ Distress tolerance**
				Distress tolerance		Commitment for the duration of the program		46.5 years (11.6)			**↑ Mindfulness**
				Emotion dysregulation				78.6			**↓ Psychological distress**
				Negative relationships				DR = 34.4			*Gambling expenses*
											*Gambling sessions*
											*ER*
^7^Cooperman et al. (2019)	USA	SUD-Nicotine (DSM-IV-TR)	SUD-Opioids (abstinence)	To field test a DBT skills training based intervention for SUD-tobacco and Opioid relapse prevention (DBT-Quit) among people in methadone treatment	Cohort (pre/post)	Lifetime smoking of 100 cigarettes or more	NR	DBT skills training	n/a	Logbook (drug and tobacco use), Urine test, *Fagerstrom Test for Nicotine Dependence*, DERS (Pre-test, 6 weeks, 12 weeks)	**↑ Attempted to stop**
						Smoking almost every day		*n* = 7			**↑ Abstinence**
						Carbon monoxide level > 8 ppm		39.0 years (14.0)			**↓ Cigarette use**
						Telephone access		100			**↓ Drug use**
						English-speaking		DR = 0.0			*ER*
						Methadone for 3 months or more					
						Not treated for SUD-Nicotine					
						Not pregnant, planning to be pregnant or breastfeeding for next 3 months					
						Wish to quit smoking in the next month					
						Accept video recording					
						18 years or older					
						No drugs on intake					
^8^Dingle and Carter (2017)	Australia	SUD-Nicotine (NR)	NR	To test the effectiveness of a new online program using personalized music listening, called "*Smoke into Sound*" for chronic smokers	Randomized controlled trial	18 years or over	NR	Smoke into Sound	Online CBT	Smoking status, *Craving Experience Questionnaire*, Confidence in managing emotions without smoking, *Fagerstrom Test for Nicotine Dependence*	**↑ Abstinence**
						Current smoker		*n* = 19	*n* = 18	(Pre-test, 6 weeks)	**↑ ER**
								40.37 years (9.64)	40.72 years (11.02)		*Craving strength*
								45	67		
								DR = 42.*1*	DR = 27.8		
									Telephone CBT		
									*n* = 18		
									41.39 years (12.73)		
									32		
									DR = 22.2		
^9^Flynn et al. (2019)	Ireland	SUD (WHO criteria)	Bipolar, personality, depression, anxiety, and mood disorders	To evaluate the effectiveness of a 24-week DBT skills-only program for individuals with comorbid emotional dysregulation and SUD	Cohort (pre/post)	Dual diagnosis	Self-harm in the last 6 months	DBT skills training	n/a	DERS, *Cork Impact of Substance Misuse Scale*	**↓ Alcohol use**
						Attending a public community-based adult drug addiction service	Severe developmental delays, cognitive impairment or learning difficulties	*n* = 64		(Pre-test, 24 weeks, 52 weeks)	**↓ Drug use**
							Active psychosis or primary diagnosis for which other treatments are recommended	NR			**↓ Dysfunctional adaptation**
								61.9			**↓ Emotional Dysregulation**
								DR = 70.3			**↓ Use of declared substances**
											*No significant change in these variables at 6-month follow-up*
^10^Ford et al. (2018)	USA	SUD-Alcohol (*Youth Adult Alcohol Problem Severity Test*)	Childhood and complex trauma	Clinical trial of an ER manual enhancing Internet-supported CBT for moderate alcohol use with college students with a history of interpersonal trauma	Randomized controlled trial	Current alcohol abuse or lifetime alcohol dependence or being in intensive treatment	Imminent danger of suicide	CBT + TARGET	CBT (standard treatment)	*Global assessment of individual needs-Short Screener alcohol use subscales* (GAIN-SS), *Negative Mood Regulation Scale*	**↓ Frequency of alcohol use**
						History of complex trauma	Inpatient psychiatric or substance abuse treatment in the past month	*n* = 13	*n* = 16	(Pre-test, 4 weeks, 8 weeks)	**↓ SUD-Alcohol**
						Self-reported current PTSD symptoms		NR	NR		**↓ PTSD symptoms**
								NR	NR		**↑ ER**
								DR = NR	DR = NR		*Excessive alcohol use*
								Total sample			
								*n* = 29			
								M(SD) = 20.1 years (1.1)			
								52			
								DR = 41.0			
^11^Frankl et al. (2019)	Sweden	SUD-Alcohol (DSM-IV)	n/a	Explore whether psychotherapy aimed at improving adaptive affective functioning and self-soothing helps:	Case study	Woman	Having one or more Axis I disorder requiring intensive treatment	Affect phobia therapy (APT)	n/a	*Timeline Followback, Penn Alcohol Craving Scale*	Carey:
				↓ alcohol consumption		18–65 years old	Current using of other drugs	*n* = 3		(Pre-test, every week for 14 weeks)	**Abstinence pre-treatment, and non-problematic use post-treatment**
				↓ comorbid psychiatric symptoms		Consent to participation	Severe Axis II disorder	50.0 years (8.72)^a^		Blood alcohol level,	**↓ Heavy drinking**
						Having an address in Stockholm County	Dementia, brain injury or severe somatic illness	100		*Affect Phobia Test*	**↓ Blood alcohol level**
						SUD-Alcohol (DSM-IV)	Do not speak Swedish	DR = 0		(Pre-test, 11 weeks)	**↓ Affect phobia**
						Have consumed alcohol for at least 5 days in the last 35 days	Currently undergoing psychotherapy				Michelle:
						No alcohol consumption in the last 7 days					*↓ Alcohol use in treatment and *↑ *at post-treatment*
						Positive screening for Affect phobia					**↓ Heavy drinking**
											**↓ Total alcohol use**
											***↑ Affect phobia***
											Mary:
											*Alcohol use in treatment*
											**↓ Heavy drinking**
											**↓ Total alcohol use**
											**↓ Blood alcohol level**
											**↓Affect phobia**
^12^Garland et al. (2019)	USA	SUD-Opioids (NR)	Chronic pain	Using Ecological Momentary Assessment as indications of the therapeutic effect of a mindfulness program (MORE) for methadone patients with pain and SUD-opioids	Controlled clinical trial	English speaking	NR	MORE (Mindfulness)	Treatment as usual	Momentary ecological assessment of craving, pain, positive affect and event-related cravings	**↓ Opioid urge**
						18 years or older		*n* = 15	*n* = 15	(2x/day for 56 days)	**↓Pain**
						Admitted on methadone maintenance treatment in the past year		47.9 years (8.7)	52.9 years (8.4)		**↓ Stress**
						Chronic non-cancer pain		53	47		**↑ Positive affect**
								DR = 6.7	DR = 13.3		**↓ Opioid wanting**
^13^Hadjiyane (2020)	USA	SUD (AUDIT and DAST-10)	NR	Study the effectiveness of a one-time intervention (CARESS) in:	Randomized controlled trial	18 years or older	Recent psychiatric hospitalization	CARESS (ER)	Treatment as usual (isometric intervention)	AUDIT, DAST-10,	*Physiological manifestations of cravings*
				Managing cravings		SUD-Alcohol or Drug (according to Audit and DAST-10)	Under the influence of alcohol or illicit/non-prescription drug	*n* = 48	*n* = 48	(Pre-test)	*Drink and drug refusal self-efficacy*
				Drug and drink refusal skill self-efficacy			Having received one of the therapeutic interventions at the outpatient facility	NR	NR	*Penn Alcohol Craving Scale*, PANAS, *Drug-Taking Confidence Questionnaire*	*Negative affect*
				Physiological responses			Suicidality or homicide risk	29.2	33.3	(Pre-test, Post-test)	
				Affect disturbance			Psychosis	DR = NR	DR = NR		
							Unable to read English without assistance	Total sample			
								*n* = 96			
								NR			
								31.25			
								DR = 4.9			
^14^Holzhauer et al. (2021)	USA	SUD-Alcohol (NR)	Depression, PTSD	In veteran women, examine the effects of Cognitive reappraisal on:	Randomized controlled trial	18 years or older	Severe mental illness	Cognitive reappraisal micro-intervention	Attention- control psychoeducation	*Timeline Followback,* AUDIT	**↓ Craving**
				Inhibitory control		AUDIT-C score of 3 or more	Brain injury or physical limitations affecting participation	*n* = 25	*n* = 25	(Pre-test)	**↑ Inhibitor control**
				Alcohol craving		Alcohol substance of choice	Current suicidal ideation	45.2 years (10.98)	46.16 years (12.28)	PANAS, *Alcohol craving questionnaire* short form revised	**↓ SUD-Alcohol severity**
						Alcohol use in the past 45 days	Pregnancy	100	100	(Pre-test, Post-stressor, Post-test)	
						Able to write and speak English		DR = 0	DR = 0		
						Served in the U.S. Military					
^15^Huang and Chen (2021)	China	SUD-Alcohol (ICD-10)	n/a	Evaluating the feasibility and efficacy of using intensive group music therapy based on ER skills to treat Chinese male inpatients with SUD-Alcohol	Controlled clinical trial	SUD-Alcohol (ICD-10)	Severe physical illness and psychotic symptoms	ER skills + Music therapy	Treatment as usual	*Craving Visual Analogue Scale* (CVAS)	*Anxiety level*
						Score ≥ 6 on the *Michigan Alcoholism Test*	Serious suicidal or self-harming tendencies	*n* = 18	*n* = 18	(Pre-test, 2 weeks)	*Sleep quality*
						Mild or no physical withdrawal symptoms	Other SUD than alcohol	41.39 years (5.08)	40.32 years (6.11)		*Alcohol craving*
						6 + years of education		0	0		*Mood*
						Informed consent to participate		TA = 33.3	TA = 0.0		
^16^Kavanagh et al. (2006)	Australia	SUD-Alcohol (DSM-IV)	n/a	To test the impact of incorporating an affective component into alcohol cues as part of the cue exposure process	Controlled clinical trial	Desire for treatment for SUD-alcohol with moderation goal	History of psychotic disorder or self-harm	CBT + Cue exposure (CE)	CBT	*Alcohol consumption report card*	**↓ Alcohol use**
						Difficulty controlling alcohol use when experiencing negative emotions	Current major depressive episode or PTSD	*n* = 52	*n* = 55	(Pre-test, Post-test, 24 weeks, 52 weeks)	*SUD-Alcohol*
						Drinking at least once when experiencing negative emotional state during baseline	Insufficient English for treatment	NR	NR	AUDIT, *Problem Drinking Questionnaire*, *Severity of Alcohol Dependence Questionnaire*, *Impaired Control Questionnaire*	*Depression*
							Current SUD other than alcohol	NR	NR	(Pre-test, 24 weeks, 52 weeks)	
							Moderate drinking contraindicated	DR = NR	DR = NR		
							Dysthymia, history of major depression or antidepressant pharmacotherapy not excluded	CBT + Emotional CE			
								*n* = 56			
								NR			
								NR			
								DR = NR			
								Total sample			
								*n* = 163			
								43.2 years (9.21)			
								56			
								DR = NR			
^17^Kooteh et al. (2020)	Iran	SUD-Opioids (NR)	NR	Compare the efficacy of combined tDCS and ER training with each separate therapy for:	Cohort (pre/post)	NR	NR	Transcranial direct current stimulation (tDCS) + ER	tDCS	*Desires for Drug Questionnaire*	tDCS + ER > tDCS or ER:
				↓ Drug craving				*n* = 18	*n* = 18	(Pre-test, Post-test)	**↓ Drug craving**
				↓Drug related thoughts and fantasies				NR	NR		**Drug related thoughts and fantasies**
								0	0		
								DR = NR	DR = NR		
									ER		
									*n* = 18		
									NR		
									0		
									DR = NR		
								Total sample			
								*n* = 54			
								M (SD) = 29.93 years (4.96)			
								0			
								DR = 18.5			
^18^Lotfalian et al. (2020)	USA	SUD-Nicotine (NR)	NR	In a controlled laboratory setting, examine the effects of a yogic breathing technique accompanied by explicit instructions on attention to breathing on:	Randomized controlled trial	18–65 years old	Pregnancy	Yogic breathing based on mindfulness	Cognitive strategy	*Fagerström Test of Nicotine Dependence*, *Minnesota Tobacco Withdrawal Scale*, Perception of nicotine withdrawal, *Urge rating scale*, PANAS, Smoking abstinence	**↓ Smoking craving**
				Smoking craving, withdrawal, negative affect		Able to read and write in English	Smoking-related health problems	*n* = 20	*n* = 20	(Pre-test, Post-test)	**↓ Perceived nicotine withdrawal**
				Mindfulness		Able to use a computer	Smoking cessation pharmacotherapy	39.55 years (12.78)	40.65 years (13.58)		**↓ Negative affect**
				Smoking behavior		Smoking 5 or more cigarettes a day for at least 1 year		50	50		**↓ Risk of smoking**
						No current smoking cessation treatment		DR = 10.0	DR = 5.0		**↓ Cigarette use**
											**↓ Acute negative effects of abstinence**
											**↓ Smoking behavior**
											*Positive affect*
									No treatment		*Mindfulness*
									*n* = 20		
									40.95 years (12.61)		
									50		
									DR = 0.0		
^19^Maffei et al. (2018)	Italy	SUD (DSM-IV)	Multiple comorbidities	In a 3-month DBT-ST program for SUD-alcohol, evaluate:	Cohort (pre/post)	Be admitted to a alcohol dependence treatment unit	Severe psychotic or cognitive disorders	DBT-ST	n/a	Consecutive days of abstinence	**↑ Days of abstinence**
				The duration of abstinence		SUD-Alcohol diagnosis (DSM-IV-TR)	Axis I disorders in active phase	*n* = 244		(Pre-test, Post-test)	**↑ ER**
				Alcohol and substance use				47.14 years (9.14)		DERS	
				Changes in ER				38.9		(Pre-test, 4 weeks, Post-test)	
				Dose effect of treatment attendance on results				DR = 35.*7*			
				Relations between abstinence and ER							
^20^Månsson et al. (2022)	Sweden	GD (DSM-5)	Depression and other psychiatric disorders	Examine the acceptability and feasibility of ER-enhanced CBT for Gambling disorder	Cohort (pre/post)	GD (DSM-5)	Manic episode and reported to have gambled during this episode	ER-enhanced group CBT	n/a	*Gambling Symptoms Assessment Scale*, DERS; *Gambling Urge* Scale,	**↓ Gambling symptoms**
				Investigate changes 12 months post-treatment on:		18 years or older	Acute psychiatric symptoms	*n* = 21		(Pre-test, Post-test, 12 weeks, 24 weeks, 52 weeks)	*ER*
				GD		Able to read and speak Swedish		36.3 years (9.0)		*Craving Experience Questionnaire for Gambling*, AUDIT,	
				Psychiatric comorbidity		Available for group sessions		19		(Pre-test, 8 weeks, 12 weeks, 24 weeks, 52 weeks)	
								DR = 43.0		Money and time spent gambling in the last week	
										(Each week of treatment, 12 weeks, 24 weeks, 52 weeks)	
^21^Pluhar (2020)	USA	IUD (interactive media)	n/a	Documenting the benefits of IUD-adapted DBT in a 15-year-old male with dysregulated use of the Internet	Case study	n/a	n/a	DBT adapted for IUD	n/a	Daily diary reporting pornography viewing, phone use at bedtime and intensity of cravings	**↑ Self-regulating behaviors**
								*n* = 1		(Each week of treatment)	**↓ Nighttime screen use**
								15.0 years (0)			**↓ Frequency of pornography use**
								0			**↑ School results**
								DR = 0			
^22^Price et al. (2019a), Price et al. (2019b)	USA	SUD (NR)	Interpersonal trauma, PTSD	To examine the longitudinal effects of Mindful awareness in body-oriented therapy (MABT) as an adjunct to the treatment of SUD in women	Controlled clinical trial	Woman	Untreated psychotic diagnosis or symptoms	MABT	Women's health education	Respiratory sinus arrhythmia, DERS, *Timeline followback*, *Penn Alcohol Craving Scale*	**↑ Interoceptive awareness**
				To promote self-care and ER		Enrolled in intensive outpatient treatment	Refusal or inability to follow treatment for the duration of the study	*n* = 93	*n* = 56	(Pre-test, 12 weeks, 24 weeks, 52 weeks)	**↑ Mindfulness skills**
						Agree to forgo manual and mind–body therapies for 3 months	Inability to give informed consent	35.0 years (NR)	36.0 years (NR)		**↓ Emotion dysregulation**
						Authorize access to medical records	Pregnancy	100	100		**↑ Days of abstinence**
						Fluent in English		DR = 21.5	DR = 17.9		**↓ Depressive symptoms**
						Able to attend the program					*Substance craving*
									Treatment as usual		
									*n* = 68		
									35.0 years (NR)		
									100		
									DR = 1.47		
^23^Ramezani et al. (2019)	Iran	SUD	NR	To determine the effect of mindfulness-based cognitive therapy (MBCT) on:	Controlled clinical trial	SUD treatment at the clinic	Absence from more than 2 sessions	MBCT	Treatment as usual	*Dependence Severity Index*, *Cognitive Emotion Regulation Questionnaire*	**↓ Severity of SUD**
				↓SUD intensity		No concurrent treatments	Refuse to continue treatment	*n* = 16	*n* = 13	(Pre-test, 5 weeks)	**↑ ER**
				↑ER			Do not use methadone	31.27 years (3.61)	35.87 years (2.23)		
				In SUD patients under methadone treatment			Being illiterate	0	0		
								DR = NR	DR = NR		
^24^Shirk et al. (2022)	USA	GD (DSM-5)	PTSD, Schizophrenia, Bipolar disorder	To illustrate the usefulness of mindfulness-based relapse prevention as a treatment for GD in 3 veterans	Case study	U.S. Army veteran	NR	Mindfulness-based relapse prevention	n/a	Gambling frequency, Frequency of cravings, Intensity of cravings, DERS—*short form*	**↓ Gambling frequency**
						Received outpatient mental health care at a veteran hospital		*n* = 3		(Pre-test, 8 weeks)	**↓ Frequency of craving**
								51.67 years (5.51)^a^			**↓ Intensity of craving**
								0			**↑ Management of urges**
								DR = NR			**↓ Impulsivity**
											**↑ ER**
											**↑ Functioning**
^25^Siu et al. (2018)	China	SUD-Ketamine (NR)	Uropathy	To analyze the outcomes of a short-term hospitalization and support program for people who abuse Ketamine	Cohort (pre/post)	16 to 30 years old	Dual diagnosis of schizophrenia, intellectual disability or organic brain disorders	Psychoeducation + psychosocial group	No treatment	Drug use, *Depression Anxiety Stress Scale*	**↑ Motivation for treatment**
						History of ketamine abuse		*n* = 84	*n* = 34	(Pre-test, 2 weeks, 13 weeks)	**↓ Anxiety**
						Agreed to participate in the 5-day hospitalization program		27.9 years (4.81)	27.6 years (5.78)		**↓ Treatment needs**
						Presence of uropathy		57.1	47.1		*Self-efficacy in drug avoidance*
						Qualified for short-term medical treatment		DR = NR	DR = NR		*Drug use*
											*Lifestyle quality*
											*Cognitive screening test*
^26^Stappenbeck et al. (2021)	USA	SUD-Alcohol (Criteria of the *National Institute on Alcohol Abuse and Alcoholism*)	NR	To evaluate the initial efficacy of a brief web-based intervention to reduce heavy drinking among college women with a history of sexual assault	Controlled clinical trial	Woman	NR	DBT, social learning theory and theory of planned behavior	No treatment	DERS; *Daily Drinking* Questionnaire,	**↓ Drinking quantity**
						18 years or over		*n* = 100	*n* = 100	(Pre-test, 2 weeks, 4 weeks, 24 weeks)	**↓ Heavy episodic drinking**
						Lifetime history of attempted or completed oral, vaginal or anal rape		21.1 years (3.6)	20.8 years (1.6)	Negative emotions, Willingness to drink	**↑ ER**
						Have had at least 2 episodes of heavy drinking in the past month		100	100	(Every day for 14 days)	**↓ PTSD symptoms**
						Have consumed an average of 7 or more standard drinks per week in the past month		DR = NR	DR = NR		
								Total sample			
								DR = 28.5			
^27^Stasiewicz et al. (2013)	USA	SUD-Alcohol (DSM-IV)	Mood and anxiety disorders	Evaluating the effectiveness of affect regulation training in treating negative affect drinking	Randomized controlled trial	Outpatient treatment of alcohol-induced SUD	Acute psychosis	CBT + affect regulation training	CBT + health and lifestyle intervention	*Timeline Followback*, *Inventory of Drug Taking Situations—Alcohol Version*	**↑ Satisfaction with treatment**
						SUD-Alcohol (according to DSM-IV)	Use of medication that may affect alcohol use	*n* = 39	*n* = 38	(Pre-test, 12 weeks, 24 weeks, 52 weeks)	**↓ Alcohol use**
						Negative affect profile for alcohol consumption	Changed mood medication in the last 3 months	NR	NR	PANAS, *Emotion Regulation Questionnaire*, DERS	**↑ Days of abstinence**
						Living within commuting distance to the program site	Other SUD except alcohol, nicotine or cannabis	NR	NR	(Pre-test, 12 weeks)	**↓ Drinks per day**
							Legally mandated to attend treatment	DR = 35.9	DR = 36.8		**↓ Drinks per occasion**
											*Heavy drinking days*
								Total sample			*ER*
								*n* = 77			
								45.7 years (11.1)			
								49.3			
								DR = 36.4			
^28^Szasz-Janocha et al. (2020)	Germany	IUD (ICD-11)	NR	Investigate the effects of PROTECT + , a CBT-based group intervention program for adolescents	Cohort (pre/post)	Subjective psychological tension due to excessive gaming or use of the Internet by teenagers or their parents	n/a	CBT-based group program	n/a	*German Video Dependency Scale*, *Compulsive Internet Use Scale—German Version*, IUD symptom severity, Time spent online, *German Questionnaire for Assessment of Emotion Regulation in Children and Adolescents*	**↓ IUD symptoms severity**
								*n* = 54		(Pre-test, Post-test, 16 weeks, 52 weeks)	**↓ Depression**
								13.48 years (1.72)			**↓ Social anxiety**
								16.7			**↓ Performance anxiety**
								DR = 29.0			**↓ School anxiety**
											**↓ General psychopathology**
											*Time spent online*
^29^Tarrega et al. (2015)	Spain	GD (DSM-5)	SUD	In men with severe GD, evaluate the impact of a serious videogame incorporated into a CBT program on:	Cohort (pre/post)	n/a	Psychiatric or neurological disorders that may interfere with game performance	CBT + Serious game	n/a	*South Oaks Gambling Screen*, *Stinchfield's Diagnostic Questionnaire for Pathological Gambling According to DSM-IV Criteria*, *State-trait Anger*	**↓ Impulsivity**
				Impulsivity traits			Medication that may affect autonomic functioning or game performance	*n* = 16		*Expression Inventory 2*	**↓ Anger expression**
				Anger expression			Current or lifetime diagnosis of Internet gaming disorders	34.8 years (6.02)		(Pre-test, Post-test)	**↓ Psychopathological symptoms**
				Emotional distress				0			
								DR = 12.5			
^30^Vinci et al. (2014), Vinci et al. (2016)	USA	SUD-Alcohol (5 + drinks on one occasion over 5 or more days in the past month)	NR	In college students with at-risk levels of alcohol use, examine the effects of a mindfulness intervention on:	Randomized controlled trial + Controlled clinical trial	AUDIT score of 6 or more	n/a	Mindfulness intervention	Search puzzle (control)	AUDIT	**Post-test**
				Negative affect		High score on at least 1 of the 2 subscales of the Questionnaire on Reasons for Drinking—Revised		*n* = 67	*n* = 66	(Pre-test)	**↑ State mindfulness**
				Alcohol urges				NR	NR	PANAS, Urge to drink	**↑ Relaxation**
				Positive affect				NR	NR	(Pre-test, Post-test, Post-affect induction)	**↓ Negative affect**
				Determine whether the type of intervention moderates the relationship between impulsivity and response to intervention				DR = 0.0	DR = 0.0		*Urge to drink*
									Relaxation intervention		(↑ Urge to drink for some individuals more impulsive for mindfulness intervention)
									*n* = 74		*No post-induction effect on affect*
									NR		
									NR		
									DR = 0		
								Total sample			
								*n* = 207			
								20.13 years (1.89)			
								76.3			
								DR = 0.0			
^31^Whiteside (2010)	USA	SUD-Alcohol (4 + /5 + drinks per occasion for women and men respectively)	NR	To evaluate a DBT-skills enhanced version of the BASICS competencies with mood-disordered heavy drinking college students	Randomized controlled trial	Binge drank at least once in past month	n/a	DBT + Motivational interviewing (DBT + BASICS)	Relaxation	*Quantity Frequency Index and Peak blood alcohol content*, *Daily Drinking Questionnaire*, DERS, *Harvard Alcohol Survey*	**↓ Alcohol-related problems**
						Drank at least weekly		*n* = 43	*n* = 53	(Pre-test, Post-test, 12 weeks)	**↓ Depression**
						Reported drinking at least "some of the times" to cope with negative emotions		NR	NR	*Rutgers Alcohol Problem Index*	**↓ Anxiety**
						Score of 14 or more on the Beck Depression or Anxiety Inventory		NR	NR	(Pre-test, 12 weeks)	**↓ Coping drinking**
								DR = 27.91	DR = 35.85		**↑ ER**
									Motivational interviewing (BASICS)		*Episodic heavy drinking*
									*n* = 49		*Maximum blood alcohol level*
									NR		
									NR		
									DR = 28.57		
								Total sample			
								*n* = 145			
								18.92 years (1.22)			
								60			
								DR = 31.03			
^32^Wilks et al. (2018)	USA	SUD-Alcohol (4 + /5 + drinks over a 2-h period for women and men respectively)	NR	To preliminary evaluate an Internet-delivered DBT skills training intervention versus a waitlist control on a sample of suicidal individuals who engage in heavy episodic drinking and experience difficulties with ER	Randomized controlled trial	Suicidal ideation in the past month	Enrolled in psychotherapy	DBT	Waiting list	AUDIT, *Timeline Followback*, DERS	**↓ Alcohol use**
						2 or more episodes of heavy drinking in the past month	Have been diagnosed with Bipolar type 1 or a psychotic disorder	*n* = 31	*n* = 29	(Pre-test, Post-test)	*ER*
						DERS score > 46	No understanding of verbal or written English	38.0 years (11.3)	37.4 years (10.1)		
						Lived in the United States	No access to a computer with Internet	66.7	72.4		
								DR = NR	DR = NR		
								Total sample			
								DR = 35.5			
^33^Witkiewitz and Bowen (2010)	USA	SUD (NR)	NR	To further examine one hypothetical mechanism of change following mindfulness-based relapse prevention	Randomized controlled trial	Fluent in English	Current psychosis, dementia, imminent suicidal risk or significant risk for withdrawal	Mindfulness-based relapse prevention	Treatment as usual	*Timeline Followback*, *Penn Alcohol Craving Scale*	**↓ Depressive symptoms**
						Completed intensive outpatient or inpatient treatment within the last 2 weeks	Inability to attend treatment	*n* = 93	*n* = 75	(Pre-test, Post-test, 8 weeks, 16 weeks)	**↓ Craving**
							Required more intensive treatment	NR	NR		**↓ Substance use**
								NR	NR		**↓ Days of use**
								DR = 22.6	DR = 32.0		
								Total sample			
								*n* = 168			
								40.45 years (10.28)			
								36.3			
								DR = 26.8			
^34^Wolitzky-Taylor et al. (2022)	USA	SUD-Cannabis (MINI neuropsychiatric interview)	Major depressive episode, Generalized anxiety disorder, Social anxiety, Panic disorder, Agoraphobia, OCD, PTSD, SUD-Alcohol or other	Comparing the preliminary efficacy of affect management therapy to CBT for SUD, for young adults with SUD-cannabis and elevated negative affect	Randomized controlled trial	18–25 years	No marked cognitive impairment	Affect management treatment	CBT	PANAS	**↓ Negative affect**
						SUD-cannabis according to MINI	No severe suicidality	*n* = 26	*n* = 26	(Pretest, each week of treatment, 6 weeks, 24 weeks)	**↓ Reactivity to negative affect**
						Score > 1 standard deviation above the norm on the Negative Affect subscale on PANAS and on either the *Anxiety Severity Index*, the *Distress Tolerance Scale* or the suppression subscale of the *Emotion Regulation Questionnaire*	No unstable manic or psychotic symptoms	22.19 years (1.67)	22.12 years (2.29)	DERS, *Emotion Regulation* Questionnaire	**↑ ER**
						No medication or stabilized on medication	Primary SUD is not cannabis	38.5	42.3	(Pre-test, 6 weeks, 12 weeks, 24 weeks)	*Cannabis use*
						Fluent in English		DR = 66.67	DR = 48.0	*Timeline Followback*, *Cannabis Abuse Screening Test*	*Problems related to cannabis use*
										(Pre-test, 12 weeks, 24 weeks)	
^35^Won and Han (2018)	South Korea	SUD-Alcohol (DSM-5)	NR	Identify the effects of a goal-oriented self-regulation program for SUD-alcohol on:	Cohort (pre/post)	SUD-alcohol (DSM-5)	Serious aggression or hostility	Goal-focused self-regulation program (GFSRP)	Treatment as usual	*Alcohol Dependence Scale—Korean version*	**↑ Abstinence self-efficacy in situations of negative affect**
				Control failure		Received specialized treatment for SUD-Alcohol	Inability to complete a questionnaire or participate	*n* = 50	*n* = 48	(Pre-test)	**↑ Optimism**
				Life goals				51.56 years (7.94)	49.77 years (9.66)	Alcohol self-regulation	*Intrinsic life goals*
				Abstinence self-efficacy				0	0	(Pre-test, 8 weeks)	
				Optimism				DR = 52.0	DR = 66.67		
^36^Yousefi et al. (2019)	Iran	SUD-Opioids (DSM-5)	NR	In people with SUD-Opioids, investigate the effects of self-control training on:	Controlled clinical trial	Man	Other psychiatric or physical disorders	CBT + assertiveness training	Waiting list	*Cognitive Emotion Regulation Questionnaire*, *Toronto Alexithymia Scale*, *Craving Beliefs Questionnaire*, *Emotional Processing Scale*	**↑ Positive ER**
				Emotional well-being		20–50 years	Absent more than once	*n* = 25	*n* = 25	(Pre-test, 10 weeks)	**↑ Emotion recognition**
				Opioids craving		History of at least 2 years of opioid use		33.25 years (7.12)	35.20 years (5.59)		**↑ Emotion processing**
						At least one withdrawal attempt		0	0		**↓ Negative ER**
						Use opium, heroin or crack		DR = 32.0	DR = 32.0		**↓ Alexithymia**
						Have at least a primary education					**↓ Opioid craving**
^37^Zargar et al. (2019)	Iran	SUD (DSM-5)	NR	To examine the effectiveness of ER therapy according to Gross' model, on:	Controlled clinical trial	Diagnosis of SUD (DSM-5) by a psychiatrist	Absent for more than 2 sessions	ER Group therapy (ERGT)	Waiting list	DERS	**↑ Marital adjustment**
				Craving		No psychological treatment for at least 1 month prior to the study	Having reused substances	*n* = 17	*n* = 17	(Pre-test, 8 weeks)	**↓ Craving**
				Emotion dysregulation		18–60 years		25.70 years (5.77)	24.85 years (2.39)		**↑ ER**
				Marital adjustment		Completion of at least grade 3 ^e^		0	0		
				in patients with SUD		Agreed to participate		DR = 11.8	DR = 11.8		
						No psychotic symptoms					
						No other psychiatric disorder					
^38^Zhuang et al. (2018)	China	GD (DSM-IV)	Depression, Anxiety	In a Chinese population of problem gamblers, examine the short-term (post-intervention) and longer-term (6 months) effects of culturally adapted CBT group treatment on:	Controlled clinical trial	Man	Severe mental illness history	CBT	Social activity group	*South Oaks Gambling Screen*, *Gambling Urge Scale*, *Gambling Activity Record*	**↓ Severity of gambling**
				Gambling-related dysfunctional thoughts		18–65 years old	Suicidal ideation or attempts in the last 3 months	*n* = 42	*n* = 42	(Pre-test, 8 weeks, 32 weeks)	**↓ Gambling-related cognitions**
				Negative psychological states		Cantonese- speaking Chinese	Had received CBT	NR	NR		**↓ Negative psychological states**
						Score of 3 or more on the *South Oaks Gambling Screen*		0	0		**↓ Money spent in the last month**
						Newly admitted to the gambling treatment service		DR = 11.9	DR = 21.4		

^a^The mean and standard deviation for age in this sample were calculated from the raw data reported in the article. ^#^Study identification number; SUD, Substance use disorder; NSRD, Non-substance-related disorder; *N*, sample size; $\overline{X }$ = mean; *SD*, standard deviation; *E*, range; *DR*, Drop-out rate; Exp; Experimental condition; Con., Control condition; NR, Not reported; IUD: Internet use disorder; EFGT, Emotion-focused group therapy; IAT, Internet Addiction Test; PANAS, Positive and Negative Affect Schedule; DSM-IV, Diagnostic and Statistical Manual of Mental Disorders, 4^e^ edition; BPD, Borderline Personality Disorder; PTSD, Post-traumatic stress disorder; BD, Bipolar Disorder; DBT, Dialectical Behavior Therapy; ER, Emotional Regulation; DERS, Difficulties in Emotion Regulation Scale; GD, Gambling disorder; SCID-I, *Structured Clinical Interview for DSM-IV axis I disorders*; SPQ, Shorter PROMIS Questionnaire; DBT-ST, Dialectical Behavior Therapy—Skills Training; GAD, Generalized anxiety disorder; OCD, Obsessive Compulsive Disorder; WHO, World Health Organization; AUDIT, Alcohol Use Disorders Identification Test; DAST-10, Drug Abuse Screening Test; ICD, International Classification of Diseases; CBT, Cognitive Behavioral Therapy

**Table 2:** Goals, modalities, providers and intervention content per study

Source ^#^Author(s) (year)	Intervention (ER techniques)	Goals	Modalities Number of sessions (Duration of a session) Frequency Modality	Providers (Setting)	Intervention content
^1^Amini et al. (2020)	EFGT	Reduce negative emotions and increase positive ones	12 sessions (2 h)	Professor (University)	**Basic Emotions**: Identify and introduce fundamental emotions. **Goals and Tools**: Understand primary and secondary goals and tools. **Experience Cycle**: Explain language’s role in emotions and introduce a memorable technique. **Trauma Work**: Guide individuals emotionally and address residual emotions. **Mental Processes**: Prevent cognitive narratives and assist with semantics. **Agency Power**: Explore the power of agency in new emotional experiences
	(NR)		Once a week		
			Group therapy		
^2^Axelrod et al. (2011)	DBT	Improve ER to control dangerous impulsive behaviors in BPD patients, such as self-harm, substance use, and other maladaptive strategies	20 sessions (90 min)	Clinicians/Professionals (Community outpatient substance abuse treatment program)	Substance use was the prioritized quality-of-life-interfering-behavior. The treatment then targets of life-threatening and treatment-interfering behaviors
	(NR)		Once a week		
			Skills group, telephone coaching as needed		
			20 sessions (1 h)		
			Once a week		
			Consultation group for the therapists		
^3^Azizi et al. (2010)	DBT, ER skills training, cognitive therapy and naltrexone	**DBT:** Identify and describe emotions, use mindfulness, reduce negative emotions, increase positive emotions, and act opposite to negative tendencies	10 sessions (90 min)	NR	In DBT, mindfulness and ER skills training. In cognitive therapy, identifying and modifying dysfunctional drug-related beliefs, and replacing them with more adaptive and functional ones
	(NR)	**CT**: Help patients build functional beliefs stronger than drug-related ones, aiming for drug abstinence	NR		
			Four modalities: group therapy, individual psychotherapy, telephone calls and consultation team meetings		
^4^Cavicchioli et al. (2019)	DBT-ST	Reduce vulnerability to distressing emotions, address substance use cues, enhance community support, and replace substance rewards with interpersonal relationships	36 sessions (180 min)	Clinicians/Professionals (Hospital)	Understanding how emotions work. Accepting emotional reactions. Reducing vulnerability to negative emotions. Increasing positive emotions. Modifying specific emotional states by acting in the opposite direction to the current emotion. Resolving problem situations that trigger emotions
	(Worksheets)		5x/week (first month) and 2x/week thereafter		
			Group therapy		
^5^Choopan et al. (2016)	ER training based on the Gross model	Teach proper management and regulation of emotions	8 sessions (120 min)	Research team (Addiction center)	**Group Familiarization:** Members and leader get acquainted, discuss goals. **Emotional Learning:** Teach emotional identification and impacts. **Vulnerability Assessment:** Evaluate emotional skills. **Emotional Change:** Prevent isolation, teach problem-solving. **Stop Rumination:** Shift focus from anxiety. **Re-evaluation:** Correct erroneous emotional evaluations. **Behavior Modification:** Learn emotional expression and behavior correction. **Goal Evaluation:** Assess and apply skills, remove obstacles
			NR		
			Group therapy		
^6^Christensen et al. (2013)	Brief DBT (Home practice of the material covered in the sessions)	Develop mindfulness, distress tolerance, ER, and interpersonal effectiveness. Improve emotional coping and reduce destructive behaviors	9 sessions (150 min)	Clinicians/Professionals (Specialist problem gambling services)	**Assessment & Orientation**: Pre-intervention assessment and “observe” mindfulness. **Mindfulness Skills**: Discuss 5 skills and set home practices. **Distress Tolerance**: Introduce radical acceptance. **Emotions**: Discuss emotions’ value and primary vs. secondary emotions. **Interpersonal Skills**: Introduce DEAR MAN and assertiveness. **Review**: Review assertiveness practice and conclude
			Once a week		
			Group therapy, individual therapy		
^7^Cooperman et al. (2019)	DBT skills training (group didactics and homework review)	Help people on methadone treatment who smoke develop DBT skills to tolerate negative emotions and cope with distress without using substances	12 sessions (90 min)	Doctorate-level group leaders (Counseling center)	**Mindfulness Skills**: Focus on being present, controlling attention, observing, participating, and non-judgment. **ER Skills**: Identify and understand emotions, reduce negative emotions, and increase positive emotions. **Distress Tolerance Skills**: Learn crisis survival strategies, tolerate painful events and emotions, and accept reality
			Once a week		
			Group therapy		
^8^Dingle and Carter (2017)	*Smoke into Sound*" musical intervention	Teach participants to identify emotions that trigger smoking and use music as a substitute for smoking’s emotion-regulating effects	6 videos (20 min)	Research team (Workplace)	**Emotion Model:** Introduce two-dimensional model; identify current and desired states. **Music Selection:** Choose music to achieve desired emotions and overcome cravings. Video 1**:** Explain quitting difficulties, smoking cues, and music as a quitting aid. **Valence-Arousal:** Explain model and smoking’s impact on feelings. **Music and Emotions:** Select meaningful music for targeted emotions. **Music Strategies:** Use music instead of smoking in various situations. **Playlist:** Create a quitting playlist
			NR		
			Online		
^9^Flynn et al. (2019)	DBT group skills training (NR)	Skills only training aims to increase goal-oriented behaviors	24 sessions (NR)	Clinicians/Professionals (Community based health addiction services)	(Module 1) Two mindfulness skills training and six distress tolerance skills training workshops. (Module 2) Two mindfulness skills training and seven ER skills training workshops. (Module 3) Two mindfulness skills training and five interpersonal effectiveness skills training workshops
			Once a week		
			Group therapy		
^10^Ford et al. (2018)	CBT + TARGET (NR)	**TARGET:** Teaches cognitive and behavioral skills for mindful awareness and stress modulation. Demystifies post-traumatic stress reactions. Provides easy methods to control stress-related impulsivity, cravings, risky behaviors, dysphoria, anxiety, and anger	8 sessions (50 min)	Graduate students (University + Online)	**TARGET Skills**: Focus on core values, recognize stress triggers, and distinguish reactive vs. adaptive emotions, beliefs, goals, and behaviors. **Stress Reactions**: Explore brain centers. **SOS Technique**: Learn focusing method. **Alarm Triggers**: Identify and manage. **Emotions**: Differentiate alarm vs. main. **Thoughts & Goals**: Distinguish alarm vs. main. **Options**: Compare alarm vs. main. **Contribution**: Use freedom steps. **Focus**: Maintain main goals with SOS
			Twice a week		
			Online individual therapy		
^11^Frankl et al. (2019)	Affect phobia therapy (weekly reports of use and craving)	Help patients function better by resolving emotional conflicts through the reduction of avoidance of adaptive and activating emotions	10 sessions (90 min first session then 45–50 min thereafter)	Research team (Public-sector addiction clinic)	Integrative therapy with psychodynamic, experiential, and behavioral components. Progressive exposure to feared affect. Preventing maladaptive avoidant response. Regulation of patient's anxiety level. Exposure to feelings
			Once a week		
			Individual therapy		
^12^Garland et al. (2019)	**MORE**: Daily 15-min mindfulness sessions with guided audio at home	Disrupt the link between chronic pain and opioid-use disorder/relapse. Increase positive responses to natural rewards to reduce cravings and addictive behaviors	8 sessions (120 min)	Clinicians/Professionals (Clinic)	Train in mindfulness, increase craving awareness and self-control, promote non-reactivity to pain, develop reappraisal skills for emotional regulation, restructure motivations for opioid use, and appreciate pleasurable events to enhance positive affectivity
			Once a week		
			Group therapy		
^13^Hadjiyane (2020)	CARESS. Communicate alternatively, release endorphin and self-soothe (NR)	Interrupt self-destructive behaviors and emotional dysregulation related to cravings and negative emotions. Promote alternative communication, release endorphins, and teach self-soothing	1 session (15–30 min)	Clinicians/Professionals (Outpatient hospital)	Drawing about emotion. Self-hugging. Soothing music as an attentional deployment and response modulation
			n/a		
			Individual therapy		
^14^Holzhauer et al. (2021)	Cognitive reappraisal micro-intervention (worksheets and manual)	Reduce negative affect	1 session (50 min)	Clinicians/Professionals (NR)	Introduce cognitive reappraisal for managing negative emotions after stress. Explain thinking traps that hinder reappraisal and sustain negativity. Teach cognitive reappraisal as an adaptive emotion regulation strategy. Guide through the cognitive reappraisal process. Practice reappraisal with a personalized stress story worksheet
			n/a		
			Individual therapy		
^15^Huang and Chen (2021)	ER skills + Music therapy (warm-up, session and homework review, listening to music, focusing on feelings)	Improve mental health by reducing negative emotions. Influence alcohol behaviors and adverse coping patterns	10 sessions (60 min)	Clinicians/Professionals (Hospital)	Musical relaxation: Guided/unguided imagery, song discussion. ER skills: Breathing, mindfulness, observing emotions, opposite action. Describe feelings: Use weather/colors, experience emotions through expressions and postures
			Five times a week		
			Group therapy		
^16^Kavanagh et al. (2006)	CBT + Cue exposure (home trials)	Repeated exposure to alcohol cues (sight, smell) without drinking. Facilitate habituation to resist triggers. Enhance treatment generalization to real settings by increasing cue salience	8 sessions (75 min)	Clinicians/Professionals (University)	**Exposure Condition**: Consume 10 g alcohol during CBT discussion. Pour, hold, look at, and sniff drink for 5 trials (3 min each), resisting drinking. Evaluate cravings, self-efficacy, and mood. Imagine non-dysphoric drinking situations. Practice at home with audio recordings (2 + times between sessions). **Emotional Exposure Condition**: Same as Exposure Condition, plus: Induce negative emotion before/during exposure (sessions 3–8). Recall increasingly negative experiences. End sessions with a positive mood
			Once a week		
			Individual therapy		
^17^Kooteh et al. (2020)	Transcranial direct current stimulation + ER training (NR)	Modify maladaptive ER strategies. Reduce drug cravings through effective emotion management. Increase flexibility and improve ER skills. Prevent relapses	8 sessions (NR)	NR (Residential drug rehabilitation center)	**Neurostim2 Stimulation**: 45-min session with 30-s rise/fall time. **Cognitive Regulation Training**:1. Stress, emotions, ART skills.2. Creating emotion, brain structures, ER strategies.3. Amygdala tension cycle, relaxation training. 4. Negative thoughts, emotional experimentation. 5. Relaxation skills, non-judgmental awareness. 6. Acceptance, observing emotions, resilience. 7. Emotional reframing, resilience technique. 8. Self-compassion practice
			NR		
			NR		
^18^Lotfalian et al. (2020)	Mindfulness-based yogic breathing (Use of techniques, self-check forms, SMS reminders)	To reduce cravings and improve abstinence	1 session (20 min)	Research team (University)	Participants were instructed to sit in a comfortable position and close their eyes. Breathing instructions were given
			n/a		
			Individual therapy		
^19^Maffei et al. (2018)	DBT skills training (Worksheets)	Replace maladaptive behaviors with goal-directed ones using four skill modules: mindfulness, ER, interpersonal effectiveness, and distress tolerance. Practice skills in daily life through homework. Reinforce abstinence with the concept of dialectical abstinence	36 sessions (180 min)	Clinicians/Professionals (NR)	**Sessions 1–2**: Prevent “abstinent violation effect.” **Sessions 3–6**: Define targets: Stop substance use. Ease withdrawal. Reduce cravings. Avoid triggers. Reduce risky behaviors. Reinforce healthy behaviors. **Sessions 7–18**: Observe emotions and urges nonjudgmentally. Improve trigger awareness and behavioral choices. **Sessions 19–24**: Tolerate stress and pain without substances. Accept reality. **Sessions 25–36**: Understand and accept emotions, reduce negative emotions, increase positive emotions, and solve problems
			Five times a week at first, then twice a week		
			Group therapy		
^20^Månsson et al. (2022)	ER-enhanced group CBT (homework, reading assignments)	To increase psychological flexibility and acceptance while pursuing goals for a valued life	8 sessions + 1 booster session (120 min)	Clinicians/Professionals (Addiction center)	1. Relapses, emergency measures, intro to gambling behavior analysis (GBA). 2. GBA, mindfulness, values, gambling consequences.3. GBA, mindfulness, emotions, psychoeducation.4. GBA, mindfulness, acceptance, problem-solving. 5. GBA, mindfulness, managing emotions. 6. GBA, mindfulness, gambling thoughts. 7. GBA, mindfulness, cognitive defusion. 8. Progress, obstacles, values, relapse prevention. 9. Antecedents, consequences of gambling urges
			Once a week		
			Individual therapy (Session 1) and group therapy thereafter		
^21^Pluhar (2020)	DBT adapted for IUD	Build skills in mindfulness, distress tolerance, ER, interpersonal effectiveness, and dialectics. Regulate interactive media use. Reduce phone use at bedtime. Reduce pornography use	14 sessions (NR)	Clinicians/Professionals (NR)	**Orientation**: Behavior education and DBT overview. **Mindfulness**: Increase awareness, reduce impulsive media use. **Distress Tolerance**: Tolerate distress with self-soothing, distraction, acceptance. **Emotion Regulation**: Identify and manage emotions, increase positive emotions. **Interpersonal Effectiveness**: Build effective relationships, improve assertiveness. **Dialectics**: Think dialectically, balance acceptance and change
	(Daily diary)		Once a week		
			Individual therapy		
^22^Price et al. (2019a); ^22^Price et al. (2019b)	Mindfulness in body therapy (Home practice)	Mindful Awareness in Body-oriented Therapy facilitates the ability to access and develop interoceptive awareness and related self-care skills	8 sessions (90 min)	Clinicians/Professionals (Community outpatient treatment clinic)	The protocol has 3 distinct stages for teaching interoceptive awareness and take-home skills. 1. Identify bodily sensations (body awareness).2. Learn and develop strategies for interoceptive awareness. 3. Develop the ability to maintain interoceptive awareness as a mindful process to facilitate the appraisal of interoceptive experiences
			Once a week		
			Individual therapy		
^23^Ramezani et al. (2019)	MABT. Mindfulness-based CBT (at-home tasks: 3-min breathing, 3x/day or in the presence of a stressor, seated meditation, preparation of activity lists)	Helping people practice biopsychological meditation. Paying attention in an intentional, moment-centered and non-judgmental way	10 sessions (120 min)	Clinicians/Professionals (Substance dependance clinic)	1. Group communication, therapeutic union, mindfulness, mindful eating. 2. Practicing thoughts/emotions, guided meditation (30–40 min). 3.Mindfulness and breathing tasks, 3-min breathing, visual/auditory meditation. 4. Present moment focus, seated meditations. 5. Permission, seated meditations. 6. Thoughts are not facts, scenario reactions, seated meditation. 7. Self-care, relapse signs discussion. 8. Training, physical checkup, reflection, feedback
			Twice a week		
			Group therapy		
^24^Shirk et al. (2022)	Mindfulness-based relapse prevention (Mindfulness at home)	Mindfulness integrated with cognitive behavioral approaches. The goal is to increase awareness, acceptance and self-compassion for urges and cravings	8 sessions (NR)	Clinicians/Professionals (Outpatient specialty mental health clinic)	1. Intro to mindfulness (M), eating exercise using senses. 2. Detailed M intro, behaviors/thoughts in relapse. 3. Triggers and cravings, surfing urges. 4. M through breathing, SOBER exercise. 5. Past situations with urges/cravings. 6. Acceptance and skillful action, psychoeducation. 7. Thoughts and relapse, non-judgmental observation. 8. Healthy lifestyles. 9. Social supports, skill implementation, tolerating discomfort
			NR		
			Group therapy		
^25^Siu et al. (2018)	Psychoeducation + psychosocial group with motivational interviewing (NR)	Address substance use and co-occurring medical, mental health, and lifestyle issues in ketamine abusers. Ensure continuity of care. Address medical and psychosocial problems through functional, psychosocial, and lifestyle interventions	NR (NR)	Clinicians/Professionals (Hospital)	Psychoeducation on ketamine abuse and functional abilities. Link between stress, emotions, and substance use. Coping with cravings using various methods and strategies. Teach and practice coping strategies (mindfulness, impulse control, assertiveness). Balance work, play, self-care, and sleep. Identify meaningful daily activities. Set short-term goals for daily engagement
			5 consecutive days		
			Group therapy		
^26^Stappenbeck et al. (2021)	Interventions based on DBT, social learning theory and theory of planned behavior (Daily evaluation logs + Home practice)	Reducing heavy drinking among college woman with a history of sexual assault	14 modules (5–10 min)	None (Online)	**Alcohol Reduction Skills**: Alcohol effects. Protective strategies. Cognitive restructuring. Drink refusal. Decision analysis. Trigger modification. Social support. **Emotion Regulation Skills**: Identify emotions, reduce negativity. Opposite action. **Distress Tolerance Skills**: Problem-solving, relaxation. Crisis approach. Decision evaluation. Reality acceptance
			Once a day for two weeks		
			Online		
^27^Stasiewicz et al. (2013)	CBT + affect regulation training (Functional analysis, self-monitoring and mindfulness and prolonged exposure homework)	ART helps patients develop their ability to regulate negative affect in a healthy way	12 sessions (90 min)	Clinicians/Professionals (NR)	Analyze antecedent events to change emotions. Behavioral analysis of drinking situations with negative affect. Train in coping skills for emotion identification and distress tolerance. Use mindfulness-based cognitive strategies. Apply exposure-based strategies to block avoidance and reduce maladaptive emotions
			Once a week		
			Individual therapy		
^28^Szasz-Janocha et al. (2020)	CBT-based group program (NR)	Manage boredom and motivation issues. Reduce procrastination, performance anxiety, and social anxiety. Promote social skills and functional emotion regulation	4 sessions (100 min)	Clinicians/Professionals (School setting)	Psychoeducation. Cognitive restructuring. Teaching life skills: problem solving, behavior modification, ER. Managing boredom and motivational problems. Reducing procrastination and performance anxiety. Reducing social anxiety and promoting social skills. Promoting ER skills
			Once a week		
			Group therapy		
^29^Tarrega et al. (2015)	CBT + Serious game (relaxing music before and after the game session)	Implement CBT strategies for total and permanent gambling abstinence. Improve problem-solving, planning, self-control, impulsive behavior control, and relaxation skills through a Serious game	16 sessions of CBT (90 min)	Clinicians/Professionals (Hospital)	**CBT**: Psychoeducation on gambling disorder. Stimulus control and response prevention. Cognitive restructuring. Reinforcement techniques. Skill training and relapse prevention. **Serious Gaming**: Adjust game difficulty based on physiological and emotional monitoring
			10 sessions of Serious game (20 min)		
			Group therapy		
			Individual therapy		
			Once a week		
^30^Vinci et al. (2014), Vinci et al. (2016)	Mindfulness (NR)	Increasing the level of state mindfulness	1 session (10 min)	Research team (College)	Focus on the present moment. Noting breathing and other sensations (sounds, images, touch, etc.). Adopt an attitude of non-judgment and acceptance
			n/a		
			Individual therapy		
^31^Whiteside (2010)	DBT + Motivational interview (personalized feedback, advice sheets)	DBT-BASIC goal is to lead to overall mood improvements based on improved ER skills	1 session (60 min)	Clinicians/Professionals (University)	Everyday topics and motivational interviewing. Open-ended questions about drinking. Comparing perceived vs. actual drinking norms. Likes, dislikes, and beliefs about drinking. Feedback from resources. Graphical comparisons of anxiety and depression. Normative feedback on emotional regulation. Questions about anxiety and depression. Impact of alcohol on mood. Reinforcing coping skills. Discussion and integration of skills. DBT skills (Mindfulness, Opposite Action)
			n/a		
			Individual therapy		
^32^Wilks et al. (2018)	DBT (worksheets, home practice, daily e-mails and SMS)	NR	8 sessions (30–50 min)	Research team (Online)	Acquire mindfulness skills. Reduce problem drinking (dialectical drinking, clear thinking, community reinforcement, burning bridges). Improve ER strategies (emotion modeling, fact checking, opposing action, problem solving, situational mastery and anticipation). Acquire distress tolerance skills
			Once a week		
			Online individual therapy		
^33^Witkiewitz and Bowen (2010)	Mindfulness-based relapse prevention (Exercises and CDs for home use)	MBRP targets negative mood and cravings to prevent relapse. It teaches relapse prevention and mindfulness meditation, helping people handle difficult situations and emotions without automatic reactions	8 sessions (120 min)	Clinicians/Professionals (NR)	Guided meditations. Experiential exercises. Discussions. Relapse prevention practices integrated with mindfulness-based skills
			Once a week		
			Group therapy		
^34^Wolitzky-Taylor et al. (2022)	Affect management therapy (NR)	AMT targets core mechanisms and processes implicated in SUD to improve treatment outcomes, by focusing on mechanisms also linked to comorbid emotional disorders	12 sessions (50 min)	Clinicians/Professionals (NR)	**Components:** Education: Avoidance, misappraisal. Mindfulness & Acceptance: Experiential avoidance, suppression, distress intolerance. Cognitive Reappraisal: Misappraisal. Problem Solving/Coping: Avoidance, negative urgency. Exposures: Behavioral/experiential avoidance, distressing stimuli. **Sessions:** 1. Motivation, problem examination, pros/cons. 2. Psychoeducation on negative affect and cannabis use, misconceptions, self-control. 3. Mindfulness for negative affect avoidance. 4. Cognitive reappraisal for catastrophic thinking. 5. Skills for negative affect, problem-solving. 6. Interoceptive exposure, fear hierarchy. 7–11. In-session exposures, debriefing.12. Treatment end, skill review, relapse prevention
			Once a week		
			Individual therapy		
^35^Won and Han (2018)	Goal-focused self-regulation program (NR)	Assist in selecting life goals. Balance “best possible self” and “worst possible self” for future-oriented thinking. Apply implementation intention	8 sessions (50 min)	Clinicians/Professionals (NR)	Sessions: 1. Self-regulation and mental health. 2. Seeking a purpose in life. 3–4. Goal orientation. 5–6. Goal-oriented ER. 7–8. Goal implementation
			Once a week		
			Group therapy		
^36^Yousefi et al. (2019)	Cognitive therapy + assertiveness training (NR)	To teach people how to manage their emotions, predict their behaviors and reward themselves to cope with craving	10 sessions (45 min)	Clinicians/Professionals (Addiction rehabilitation center)	1.Organizing training groups and planning sessions. 2. Prioritizing treatment goals. 3. Teaching self-assessment skills. 4. Self-study and observation. 5.Teaching self-reinforcement methods. 6. Teaching self-guided training. 7. Teaching self-modeling. 8. Identifying craving stimulants. 9. Teaching problem-solving techniques. 10. Reviewing and reassessing treatment plans
			Once a week		
			Group therapy		
^37^Zargar et al. (2019)	ER Group therapy (NR)	Reduce cravings and marital dissatisfaction. Recognize and regulate emotions. Overcome obstacles to and accept positive emotions. Identify and modify maladaptive and adaptive ER strategies	8 sessions (60 min)	Clinicians/Professionals (Hospital)	**Emotions & Cravings**: Types of emotions, craving as a negative event, recognizing triggers. **Emotional Issues**: Discussing emotional aspects, monitoring personal emotions. **ER Strategies**: Introducing and identifying strategies, preventing isolation, improving problem-solving. **Conflict Resolution**: Interpersonal and marital conflict resolution, active listening, expressing emotions. **Obsessive Thinking**: Managing obsessive thinking and worry. **Emotional States**: Effects of emotions, teaching reappraisal. **Inhibiting Emotions**: Identifying and modifying inhibition methods. **Review & Evaluation**: Reviewing content, evaluating therapeutic objectives
			Once a week		
			Group therapy		
^38^Zhuang et al. (2018)	CBT (NR)	NR	8 sessions (180 min)	Clinicians/Professionals (Counseling center)	1–2. Enhance motivation, identify triggers, recognize physiological gambling responses. 3–5. Increase awareness of cognitive distortions, examine behavioral responses. 6–7. Recognize negative emotions, develop strategies to manage them. 8. Consolidate gains, provide relapse prevention info
			Once a week		
			Group therapy		

NR, not reported; IUD: internet use disorder; EFGT, emotion-focused group therapy; IAT, internet addiction test; PANAS, positive and negative affect schedule; DSM-IV, diagnostic and statistical manual of mental disorders, 4^e^ edition; BPD, borderline personality disorder; PTSD, post-traumatic stress disorder; BD, bipolar disorder; DBT, dialectical behavior therapy; ER, emotional regulation; DERS, difficulties in emotion regulation scale; GD, gambling disorder; SCID-I, *Structured Clinical Interview for DSM-IV axis I disorders*; SPQ, shorter PROMIS questionnaire; DBT-ST, dialectical behavior therapy—skills training; GAD, generalized anxiety disorder; OCD, obsessive compulsive disorder; WHO, World Health Organization; AUDIT, alcohol use disorders identification test; DAST-10, drug abuse screening test; ICD, international classification of diseases; CBT, cognitive behavioral therapy

When based on a theoretical model (*n* = 31), 81.6% of the interventions are second- or third-wave cognitive-behavioral approaches. These included dialectical behavior therapy (*n* = 12; 31.6%), mindfulness (*n* = 7; 18.4%), CBT (*n* = 6; 15.8%), or various programs prioritizing ER (*n* = 6; 15.8%). Other interventions (*n* = 7; 18.4%) integrated a variety of practices. In 63.2% of the studies, the primary goal of the interventions was to enhance ER, spanning from improved identification of emotions to mastery of emotion management (see Table 1).

The number of sessions varied from one to thirty-six, with over a third (*n* = 13; 34.2%) offering eight sessions. Six of the 38 interventions (15.8%^8,13,14,18,30,31^) provided a single session. Sessions ranged from 10 min to three hours, with two hours (18.4%) being the most common. The interventions offered sessions weekly (81.6%), bi-weekly (10.5%), or daily (7.9%). Sessions were provided individually (39.5%; *n* = 15), in groups (39.5%; *n* = 15), or by combining these two modalities (18.4%; *n* = 7). One study did not describe the modality. While 89.5% (*n* = 34) of interventions were conducted in person, 10.5% (*n* = 4^8,10,26,32^) were carried out online (see Table 1).

### Categorization According to ER Intervention Content

The studies were categorized based on the content of the intervention provided and the percentage of ER sessions (see Table 2). Some of the most used techniques are presented to illustrate each category.

*Predominantly ER* (*n* = *24*, 63.2%)*.* Nineteen interventions are inspired by dialectical behavior therapy^2,3,4,6,7,9,15,19,21,31,32^, mindfulness^12,18,22,23,24,30,33^, or emotion-focused therapy^1^. Three studies developed their program using theoretical models of ER^5,17,37^. One study had three complementary ER interventions^13^ and one had a four-session thought reappraisal technique^14^. These interventions encompass a range of techniques, including education on emotions and coping strategies, as well as mindfulness practice. Most of these studies (75%) recommended mindfulness exercises for the various bodily sensations and emotions that arise while adopting an attitude of non-judgment and acceptance^2,3,4,6,7,9,12,15,18,19,21,22,23,24,30,31,32,33^. Almost half (41.7%) of these studies worked on identifying and describing a range of emotions by offering information or encouraging sharing^1,4,5,6,7,15,17,19,21,37^. Eleven interventions (45.8%) use behavior analysis (ABC model). Six interventions (25.0%^1,5,13,15,21,37^) incorporated emotional expression, and four studies (16.7%^5,12,14,37^) presented an intervention teaching cognitive reappraisal, which involves generating less negative alternative interpretations.

*Partially ER* (*n* = 8, 21.1%)*.* These interventions treated SUD and addictive disorders using traditional CBT techniques (e.g., behavioral analysis, exposure hierarchy) with the addition of emotional-focused techniques. Six of these interventions (75.0%^8,10,20,26,27,38^) offered psychoeducation on emotions and their function, to foster their identification. Mindfulness of emotions and internal sensations (37.5%^20,27,34^), and strategies for managing negative emotions and exposure were the other strategies reported (50.0%^20,27,34,38^).

*Contribution in ER not measurable* (*n* = *6*, 15.8%). Three articles presented an emotion-targeted intervention with insufficient detail, making it impossible to assess the work done in terms of ER^25,28,36^. Three studies only briefly described the intervention. One study aimed at exploration, focus, and exposure to the feared emotion^11^. Another study involved exposure to situations that trigger negative emotions in the presence of alcohol^16^. The third study promoted awareness of one's emotional state using a serious video game^29^.

### Effectiveness of Interventions

Most studies (*n* = 29; 76.3%) reported statistically significant effects on symptomology. However, it is important to note that the design of these studies did not consistently incorporate a control group (refer to Table 1). Specifically, 12 studies are cohort studies^2,4,6,7,9,17,19,20,25,28,29,35^, while three are case studies^11,21,24^. Studies reported reductions in the number of diagnostic criteria, frequency of consumption or use (*n* = 13; 34.2%^1,2,7,8,9,10,19,20,22,27,29,31,33^), craving, desire, and desire-related beliefs (*n* = 7; 18.4%^5,12,14,17,18,36,37^) or severity of addictive disorders (*n* = 4; 10.5%^4,23,28,38^). One study demonstrated a decrease in cannabis use after the intervention, similar to the control group^34^. Three case studies indicated a reduction in episodes of alcohol abuse and cravings^11^, less time spent on late-night phone calls, decreased consumption of pornography^21^, and a decrease in gambling frequency, as well as improved management of gambling cravings^24^.

Five studies showed no statistically significant effects on symptomology following intervention. They assessed desire for alcohol (*n* = 3^13,15,30^), number of sessions and money spent gambling^6^, frequency and duration of drug use^25^ as well as physiological responses related to desire for alcohol and self-efficacy in refusing drug and alcohol use^13^. A small sample size, a lack of objective measures and an uncontrolled confounding variable (creating a safety plan for dealing with suicidal thoughts may have caused stress) are reasons cited by the authors to explain these results.

Many of the studies (*n* = 28; 73.7%) assessed efficacy on at least one ER measure (see Table 1). The *Difficulties in Emotion Regulation Scale* (DERS; 50.0%) and the *Positive and Negative Affect Scale* (PANAS; 25.0%) were the most widely used validated instruments. Sixteen of the 28 studies found statistically significant post-intervention effects. These included a decrease in ER deficit (*n* = 10; 35.7%^2,3,4,9,19,22,26,31,34,37^), a decrease in negative emotions (*n* = 4; 14.3%^1,18,30,34^) or an increase in positive emotions (*n* = 2; 7.1%^1,12^), an improvement in positive cognitive ER strategies and a decrease in negative cognitive ER strategies (*n* = 2; 7.1%^23,36^). One study showed a reduction in alexithymia and an improvement in emotional processing (3.6%^36^). There was also an improvement in the tendency to regulate emotions through cognitive reappraisal (*n* = 1; 3.6%^34^). Two interventions influenced expectations for managing negative mood states^10^ and reducing negative emotions^27^. However, these were equivalent to those of the control group. Two case studies showed a reduction in ER deficit (*n* = 1; 3.6%^24^) or an improvement in affective phobia in two of their three participants (*n* = 1; 3.6%^11^).

Five studies (17.9%) reported no statistically significant effect on their ER measure. They evaluated negative affect^13^ or ER deficit (*n* = 4^6,7,20,32^). Two of the 38 studies (5.3%^6,13^) did not find a statistically significant effect on symptomology or ER.

### Risk of Study Bias

**Fig. 2:**
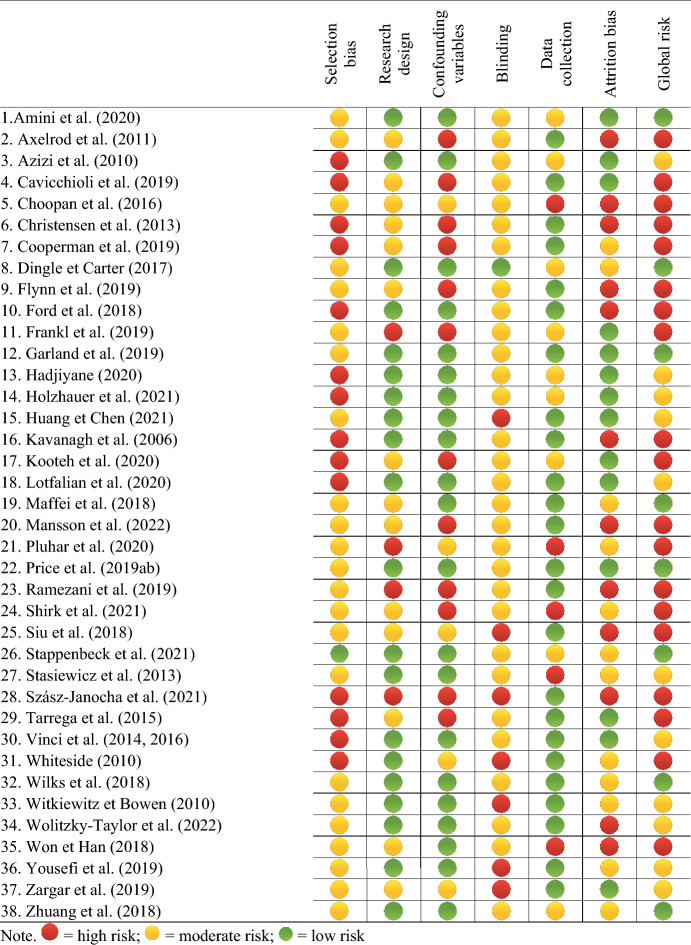
Risk of bias assessment for each study

Figure 2 visually represents the risk assessment of bias. Most of the studies (79.0%) were rated as having an overall quality of low (high risk) to moderate (moderate risk).

## Discussion

The objective of this study was to conduct a systematic review of psychological treatments designed to enhance ER in individuals with SUD or addictive disorders. To this end, the systematic review documented the content of interventions for ER related to addictive disorders and their effectiveness.

The results indicate that the interventions are effective in reducing both SUD and addictive disorders, including gambling, video games, and Internet use. Some interventions demonstrate favorable behavioral (e.g., reduced frequency of use) or cognitive (e.g., reduced craving beliefs) outcomes, supporting the conclusions of meta-analyses in the context of multiple behavioral addictive disorders (Goslar et al., 2020). The interventions identified were largely effective in improving ER, from attenuation of ER deficits to an increased tendency to use an adapted ER strategy. It should be noted that these improvements are not universal across all interventions. Two studies have demonstrated a lack of statistically significant effect on ER, although they have observed a reduction in symptomology. These findings provide support for the hypothesis that addressing ER, even in a partial manner, could prove beneficial in SUD and addictive disorders treatment. The optimal number of sessions of ER content to produce effects remains to be documented, as does the type of individuals who might best benefit from it. One such group is the emotionally vulnerable gamblers identified by Blaszczynski and Nower (2002) in their typological model.

Despite most interventions demonstrating favorable outcomes for both symptomology and ER, the question of whether these outcomes are attributable to the intervention itself remains challenging. Many studies employed research protocols that lack control groups, which constrains our capacity to ascertain the causal relationships between observed effects (Glass et al., 2013). Furthermore, numerous studies were susceptible to substantial biases. Multiple sources of bias were identified, often present in non-pharmaceutical psychological treatment studies. Two examples are the lack of statistical control for dropouts and participants not being blinded to their conditions (Boutron et al., 2007). While the data collection methods were generally well-controlled by using measurement instruments with strong psychometric properties, the fidelity of these instruments was not always reported. On the other hand, blinding and selection bias were typically the least well-controlled factors, likely due to the inclusion of clinical and intervention samples without a control group. All these biases may lead to either underestimating or overestimating treatment effects (Zaugg et al., 2014). In addition, it is impossible to determine the ER intervention's specific impact without comparing it to a standard treatment that does not include an ER component. Only three of the studies reviewed made this comparison, and their results indicate improvements in symptomology (e.g., fewer days of use) and, in two out of three studies, in ER. Nevertheless, 63% of the studies reviewed presented an intervention with a predominantly emotional component, indicating that interventions based largely on ER are sufficient to produce positive effects on ER and reduce symptomatology. Future reviews employing only robust randomized controlled trial designs will elucidate whether incorporating an ER intervention is more efficacious than standard treatment. It is important to note that no meta-analysis was conducted to provide a more precise estimate of effects. A meta-analysis would allow for more robust conclusions and help detect subtle effects that might not be apparent in individual studies. Nevertheless, the use of ER in the treatment of SUD and addictive disorders appears to be a practice that should not be overlooked.

Regarding the robustness of the effects observed, it is noteworthy that most studies employ validated instruments to assess ER, enhancing the results' reliability. Conversely, some studies employ non-validated measures designed to assess a specific aspect of ER (e.g., negative emotions) using a limited number of items (between 1 and 5). As a result, the interpretation of these results should be approached with caution.

The array of treatment programs for SUD and addictive disorders with an emotional component is notable. Interventions influenced by or rooted in third-wave CBT, such as mindfulness-based approaches, are increasingly prevalent. This finding is consistent with the existing evidence on the efficacy of these interventions, particularly in the treatment of eating disorders, psychotic disorders, or addictive disorders (Linardon et al., 2019; Louise et al., 2018; Sancho et al., 2018).

The assessment of ER in the content of the interventions listed, based on the number of sessions, does not allow for the qualification of the interventions and the work carried out. For instance, a brief intervention (15–30 min) comprising drawing, physical affection, and musical accompaniment while cultivating mindfulness exhibits no discernible beneficial impact, despite its predominantly ER orientation. In contrast, regardless of the categorization of the intervention, the identification of one’s emotions or the attention paid to one’s inner world through the description of one’s feelings appears to be the fundamental aspect of emotional work. Unfortunately, the precise process used to achieve this is not always accessible for examination. A more detailed description of the treatments or access to the material would allow for a more comprehensive examination of the content of interventions with an ER component and facilitate comparison. Further research is required to facilitate a comparative analysis of the quality of emotional interventions within the context of SUD or addictive disorders. Additionally, it is necessary to determine the optimal content and approach to provide quality emotional support.

It was not surprising to find significantly more literature on ER for SUD than for behavioral addictive disorders. In comparison to SUD, fewer interventions focus on ER for GD, IA, or IGD. One potential explanation for this discrepancy is the high comorbidity between SUD and personality disorders (Kienast et al., 2014). Considering the efficacy and esteem in which dialectical behavioral therapy is held as a treatment for personality disorders (Lee et al., 2015), particularly given the emotional deficiencies associated with such conditions, the rationale for employing it in the context of SUD appears sound. One other reason may be that cognitive interventions remain crucial in addressing behavioral addiction disorders, particularly for GD. This focus on cognitive approaches (Fortune & Goodie, 2012; Ladouceur et al., 1998) seems to be reflected in the limited number of studies exploring emotional work. Further research is required to optimize the integration of cognitive and emotional components in the treatment of addictive disorders.

While the identified interventions yielded promising outcomes, it is imperative to exercise caution when interpreting these results, given the considerable risk of bias in the studies. These findings can be attributed to several sources of bias, including the lack of statistical control for dropouts and the fact that participants were not blinded to conditions. Furthermore, despite the consultation of grey literature, publication bias may contribute to an over-representation of studies demonstrating the effectiveness of interventions (Ferguson & Brannick, 2012).

### Strengths and Limits

This review has several notable strengths. Documenting the use of ER in treatments for substance and non-substance-related disorders enhances our overall understanding of the effects of these interventions. This is particularly pertinent given the paucity of literature on behavioral addictive disorders. Furthermore, this study is the sole investigation to date to have quantified ER sessions in psychological interventions for SUD and addictive disorders. This review also has limits. It was determined that it would be preferable to avoid imposing restrictions on the study's design or the characteristics of the sample to gain a comprehensive overview of addictive disorders interventions that include an ER component. The presence of comorbidities enhances the external validity of the results, as they are more common than not (Huynh et al., 2020). Conversely, no studies were excluded based on quality assessment. The paucity of rigorous studies identified thus constrains the scope of our conclusions.

### Implications for Future Research

Additional research is essential to identify the optimal content and structure of ER interventions that yield significant effects and to determine which individuals would benefit most from them. Future reviews that utilize only well-designed randomized controlled trials would enhance our understanding of whether the inclusion of an ER intervention is more effective than standard treatment. Furthermore, conducting a meta-analysis would facilitate more definitive conclusions regarding its efficacy.

## Conclusion

This systematic review emphasizes the extensive body of literature on psychological therapies aimed at enhancing ER in individuals with SUD, as opposed to those with behavioral addictive disorders. Additionally, it highlights the need for further research into treatments that improve ER in individuals with behavioral addictive disorders. Most of the studies presented intervention content that focused primarily on ER, with a substantial number being based on third-wave CBT. The interventions showed meaningful effects on symptomatology and ER; however, caution is warranted regarding the research designs used. These findings support the role of ER in therapies for SUD and addictive disorders. Future research could clarify which essential ER components are most important to target and identify individuals who would benefit most from increased ER content in psychotherapy for SUD and addictive disorders.

## Supplementary Information

Below is the link to the electronic supplementary material.

Supplementary file1 (PDF 388 KB)

## Data Availability

Data sharing is not applicable (review).
