# Supplementary material for: Emotional Regulation in Substance-Related and Addictive Disorders Treatment: A Systematic Review
Source: J Gambl Stud. 2025 Feb 7;41(2):353–448. doi: 10.1007/s10899-024-10366-8 (PMC12116982; doi:10.1007/s10899-024-10366-8)
Supplement: Supplementary file 1 — Supplementary file1 (PDF 388 KB) [file 10899_2024_10366_MOESM1_ESM.pdf]

PsycINFO (OVID)

Database : no limit
Period of coverage: 1806 to January Week 4 2022
Update frequency: daily
Date: January 31, 2022

Concepts	#	Search Queries	Results (on 2022/01/31)
**1st Emotional regulation**	1.	(affect$ adj3 control$).ti,ab,id	5340
	2.	(affect$ adj3 regulation$).ti,ab,id	4651
	3.	(affect$ adj3 response$).ti,ab,id	12 608
	4.	alexithymia/	3221
	5.	alexithymia.ti,ab,id	4 318
	6.	emotional adjustment/	16 799
	7.	(emotion$ adj3 adjust$).ti,ab,id	4598
	8.	"emotion$ awareness".ti,ab,id	1262
	9.	emotional control/	3997
	10	(emotion$ adj3 control$).ti,ab,id	7532
	11	emotional disturbances/	10 334
	12	(emotion$ adj3 disturbance$).ti,ab,id	5778
	13	(emotion$ adj3 dysregulation$).ti,ab,id	3762
	14	emotional instability/	544
	15	(emotion$ adj3 instabilit$).ti,ab,id	1114
	16	"emotion$ manag$t".ti,ab,id	0
	17	(emotion$ adj3 reactivit$).ti,ab,id	3746
	18	emotion recognition/	3199
	19	(emotion$ adj3 recognition).ti,ab,id	5768
	20	emotional regulation/	12 708
	21	(emotion$ adj3 regulation).ti,ab,id	20 573
	22	emotional responses/	18 239
	23	"emotion$ response$".ti,ab,id	11 355
	24	emotional stability/	1353
	25	(emotion$ adj3 stabilit$).ti,ab,id	3156
	26	emotional states/	37 926
	27	(emotion$ adj3 state$).ti,ab,id	13 689

emotional regulation combined	28	or/1-27	159 264
**2nd Dependences**	29	alcohol abuse/	18 803
	30	(alcohol adj3 (misus$ or abus$ or addict$ or dependenc$ or disorder$ or bing$ or problem$)).ti,ab,id	49 925
	31	alcohol drinking patterns/	25 154
	32	alcoholism/	30542
	33	(alcoholic or alcoholism).ti,ab,id	31 869
	34	binge drinking/	2838
	35	computer usage/	863
	36	(computer$ adj3 (misus$ or abus$ or addict$ or dependenc$ or disorder$ or compuls$ or obsessi$ or excessive or overus$ or problem$)).ti,ab,id	1537
	37	(cyberaddiction or "cyber addiction").ti,ab,id	18
	38	((constant or hyper or permanent) adj3 connectivity).ti,ab,id	178
	39	designer drugs/	191
	40	digital gaming/	2382
	41	((digital or electronic or internet or online) adj3 (abus$ or addict$ or bet or bets or betting or betted or bettor or bettors or bing$ or dependenc$ or overus$ or misus$ or disorder$ or compuls$ or obsessi$ or excess$ or pathologic$ or problem$)).ti,ab,id	6372
	42	drinking behavior/	1962
	43	((drink$ or drank or drunk) adj3 (bing$ or misus$ or abus$ or addict$ or dependenc$ or disorder$ or problem$)).ti,ab,id	12155
	44	drug abuse/	48415
	45	drug addiction/	11 780
	46	drug dependency/	13 081
	47	drug usage/	21 178
	48	(drug$ adj3 (use$ or usage or misuse$ or abus$ or addict$ or depedenc$ or disorder$ or problem$)).ti,ab,id	87 914
	49	gambling/	4305

	50	gambling disorder/	5073
	51	(gambl$ adj3 (addict$ or compuls$ or obsessi$ or dependenc$ or disorder$ or excessi$ or problem$ or pathologic$)).ti,ab,id	6931
	52	impulse control disorders/	995
	53	internet addiction/	3137
	54	internet usage/	2748
	55	((mobile or smartphone$ or chat) adj3 (abus$ or addict$ or dependenc$ or overus$ or compuls$ or obsessi$ or excess$ or patholog$ or disorder$ or problem$)).ti,ab,id	1054
	56	(("social media" or "social network$") adj3 (abus$ or addict$ or dependenc$ or excess$ or overus$ or compuls$ or obsessi$ or problem$ or disorder$)).ti,ab,id	898
	57	"substance use disorder"/	9287
	58	"substance related and addictive disorders"/	16
	59	(substance$ adj3 (use$ or usage or misuse$ or abus$ or addict$ or dependenc$ or disorder$ or problem$)).ti,ab,id	84 351
	60	toxicomania/	24
	61	toxicoman$.ti,ab,id	187
	62	wager$.ti,ab,id	509
Dependences combined	63	or/29-62	258 885
**3rd Treatments**	64	"acceptance and commitment therapy"/	2193
	65	addiction treatment/	1944
	66	cognitive behavior therapy/	21959
	67	cognitive therapy/	13 754
	68	dialectical behavior therapy/	1567
	69	intervention/	75 745
	70	intervention$.ti,ab,id	433 527
	71	"mental health care".ti,ab,id	15 569
	72	mindfulness/	11 282
	73	mindfulness.ti,ab,id	16 297
	74	mindfulness based interventions/	2005
	75	(program or programs$ or programme or programmes).ti,ab,id	410 086

	76	psychotherapy/	55 854
	77	psychotherap$.ti,ab,id	125 460
	78	therap$.ti,ab,id.	437 745
	79	treatment/	80 786
	80	treatment$.ti,ab,id	683 337
Treatments combined	81	or/64-80	1 481 382
**FINAL SEARCH** all concepts **combined**	82	28 and 63 and 81	3303
	83	82 and 2000:2022.(sa_year).	2722

MEDLINE (OVID)

Database : Unlimited
Period of coverage: 1946 to January 31, 2022
Update frequency: daily
Date: January 31, 2022

Concepts	#	Query	Results (on January 31, 2022)
1st **Emotion regulation**	1.	(affect$ adj3 control$).ti,ab,kw	11 100
	2.	(affect$ adj3 regulation$).ti,ab,kw	6998
	3.	(affect$ adj3 response$).ti,ab,kw	30783
	4.	alexithymia.ti,ab,kw	3 459
	5.	emotional adjustment/	938
	6.	(emotion$ adj3 adjust$).ti,ab,kw	1702
	7.	"emotion$ awareness".ti,ab,kw	707
	8.	(emotion$ adj3 control$).ti,ab,kw	5102
	9.	(emotion$ adj3 disturbance$).ti,ab,kw	2709
	10.	(emotion$ adj3 dysregulation$).ti,ab,kw	2788
	11.	(emotion$ adj3 instabilit$).ti,ab,kw	740
	12.	"emotion$ manag$t".ti,ab,kw	0
	13.	(emotion$ adj3 reactivit$).ti,ab,kw	2770
	14.	(emotion$ adj3 recognition).ti,ab,kw	5074
	15.	emotional regulation/	1485
	16.	(emotion$ adj3 regulation).ti,ab,kw	12 702

	17.	"emotion$ response$".ti,ab,kw	6975
	18.	(emotion$ adj3 stabilit$).ti,ab,kw	1193
	19.	(emotion$ adj3 state$).ti,ab,kw	9079
Emotion regulation combined	20.	or/1-19	95 268
2nd Dependences	21.	(alcohol adj3 (misus$ or abus$ or addict$ or dependenc$ or disorder$ or bing$ or problem$)).ti,ab,kw	58 033
	22.	alcoholics/	865
	23.	alcoholism/	77 968
	24.	(alcoholic or alcoholism).ti,ab,kw	84 131
	25.	alcoholic beverages/	8032
	26.	alcohol drinking/	71 921
	27.	alcohol-related disorders/	5 572
	28.	(gambl$ adj3 (addict$ or compuls$ or obsessi$ or dependenc$ or disorder$ or excessi$ or problem$ or pathologic$)).ti,ab,kw	5079
	29.	binge drinking/	2 257
	30.	compulsive behavior/	3 231
	31.	(computer$ adj3 (misus$ or abus$ or addict$ or dependenc$ or disorder$ or compuls$ or obsessi$ or excessive or overus$ or problem$)).ti,ab,kw	1801
	32.	(cyberaddiction or "cyber addiction").ti,ab,kw	17
	33.	((constant or hyper or permanent) adj3 connectivity).ti,ab,kw	218
	34.	designer drugs/	1738
	35.	((digital or electronic or internet or online) adj3 (abus$ or addict$ or bet or bets or betting or betted or bettor or bettors or bing$ or dependenc$ or overus$ or misus$ or disorder$ or compuls$ or obsessi$ or excess$ or pathologic$ or problem$)).ti,ab,kw	6662
	36.	"Disruptive, Impulse Control, and Conduct Disorders"/	2629
	37.	drinking behavior/	6853

	38.	((drink$ or drank or drunk) adj3 (bing$ or misus$ or abus$ or addict$ or dependenc$ or disorder$ or problem$)).ti,ab,kw	12545
	39.	drug misuse/	257
	40.	drug seeking behavior/	1571
	41.	(drug$ adj3 (use$ or usage or misuse$ or abus$ or addict$ or depedenc$ or disorder$ or problem$)).ti,ab,kw	232 486
	42.	gambling/	6267
	43.	illicit drugs/	12 025
	44.	impulsive behavior/	9 139
	45.	internet addiction disorder/	451
	46.	"Internet Use"/	284
	47.	((mobile or smartphone$ or chat) adj3 (abus$ or addict$ or dependenc$ or overus$ or compuls$ or obsessi$ or excess$ or patholog$ or disorder$ or problem$)).ti,ab,kw	1439
	48.	online social networking/	305
	49.	(("social media" or "social network$") adj3 (abus$ or addict$ or dependenc$ or excess$ or overus$ or compuls$ or obsessi$ or problem$ or disorder$)).ti,ab,kw	849
	50.	street drugs/	12 025
	51.	substance related disorders/	101 003
	52.	(substance$ adj3 (use$ or usage or misuse$ or abus$ or addict$ or dependenc$ or disorder$ or problem$)).ti,ab,kw	81 808
	53.	toxicoman$.ti,ab,kw	343
	54.	video games/	6507
	55.	wager$.ti,ab,kw	380
Dependences combined	56.	or/21-55	565 327
3rd Treatments	57.	"Acceptance and Commitment Therapy"/	637
	58.	cognitive behavioral therapy/	28 377
	59.	dialectical behavior therapy/	177
	60.	intervention$.ti,ab,kw	1 160 709

	61.	"mental health care".ti,ab,kw	13 362
	62.	mindfulness/	4928
	63.	mindfulness.ti,ab,kw	10 105
	64.	(program or programs$ or programme or programmes).ti,ab,kw	869 281
	65.	psychotherapy/	56 154
	66.	psychotherap$.ti,ab,kw	49 952
	67.	psychotherapy brief/	3641
	68.	psychotherapy group/	14 348
	69.	psychotherapy multiple/	700
	70.	psychotherapy rational emotive/	197
	71.	therap$.ti,ab,kw	3 108 474
	72.	treatment$.ti,ab,kw	4 987 882
Treatments combined	73.	or/57-72	7 955 667
**FINAL SEARCH** all concepts **combined**	74.	20 and 56 and 73	2258
	75.	74 and 2000:2022.(sa_year).	2054

EMBASE
Database limited > Embase
Period of coverage: 1947 to present
Update frequency: daily
Date: January 31, 2022

Concepts	#	Query	Results (on January 31, 2022)
1st Emotional regulation	#1	(affect* NEAR/3 control*):ti,ab,kw	14 198
	#2	'affect regulation'/de	12
	#3	(affect* NEAR/3 regulation*):ti,ab,kw	8 666
	#4	(affect* NEAR/3 response*):ti,ab,kw	36 626
	#5	'alexithymia'/de	4 029
	#6	alexithymia:ti,ab,kw	4 598
	#7	(emotion* NEAR/3 adjust*):ti,ab,kw	2 234
	#8	'emotional awareness'/de	17
	#9	"emotion* awareness":ti,ab,kw	929

	#10	(emotion* NEAR/3 control*):ti,ab,kw	6 574
	#11	(emotion* NEAR/3 disturbance*):ti,ab,kw	3 848
	#12	'emotion dysregulation'/de	37
	#13	(emotion* NEAR/3 dysregulation*):ti,ab,kw	3 630
	#14	(emotion* NEAR/3 instabilit*):ti,ab,kw	1 106
	#15	'emotion* manag*t':ti,ab,kw	549
	#16	'emotional reactivity'/de	57
	#17	(emotion* NEAR/3 reactivit*):ti,ab,kw	3 523
	#18	'emotion recognition'/de	135
	#19	'emotion recognition deficit'/de	12
	#20	(emotion* NEAR/3 recognition):ti,ab,kw	6 570
	#21	'emotion regulation'/de	2 566
	#22	(emotion* NEAR/3 regulation):ti,ab,kw	16 329
	#23	'emotion* response*':ti,ab,kw	8 375
	#24	(emotion* NEAR/3 stabilit*):ti,ab,kw	1 500
	#25	(emotion* NEAR/3 state*):ti,ab,kw	11 562
emotional regulation combined	#26	#1 OR #2 OR #3 OR #4 OR #5 OR #6 OR #7 OR #8 OR #9 OR #10 OR #11 OR #12 OR #13 OR #14 OR #15 OR #16 OR #17 OR #18 OR #19 OR #20 OR #21 OR #22 OR #23 OR #24 OR #25	118 744
**2nd Dependences**	#27	'alcohol abuse'/de	31 753
	#28	(alcohol NEAR/3 (misus* OR abus* OR addict* OR dependenc* OR disorder* OR bing* OR problem*)):ti,ab,kw	85 479
	#29	((drink* OR drank OR drunk) NEAR/3 (bing* OR misus* OR abus* OR addict* OR dependenc* OR disorder* OR problem*)):ti,ab,kw	17 251
	#30	'alcoholism'/de	135 892
	#31	(alcoholic OR alcoholism):ti,ab,kw	128 692
	#32	'drug abuse'/de	54 123
	#33	'drug abuse pattern'/de	1 110
	#34	'drug dependence'/de	64 059
	#35	'designer drug'/de	1 816
	#36	'multiple drug abuse'/de	1 700
	#37	(drug* NEAR/3 (use* OR usage OR misuse* OR abus* OR addict* OR depedenc* OR disorder* OR problem*)):ti,ab,kw	329 601
	#38	'substance abuse'/de	55 478
	#39		108 709

		(substance* NEAR/3 (use* OR usage OR misuse* OR abus* OR addict* OR dependenc* OR disorder* OR problem*)):ti,ab,kw	
	#40	toxicoman*:ti,ab,kw	529
	#41	(gambl* NEAR/3 (addict* OR dependenc* OR disorder* OR compuls* OR obsessi* OR excessi* OR problem* OR pathologic*)):ti,ab,kw	6 328
	#42	'impulse control disorder'/de	3 940
	#43	'computer addiction'/de	263
	#44	(computer* NEAR/3 (misus* OR abus* OR addict* OR dependenc* OR disorder* OR compuls* OR obsessi* OR excessive OR overus* OR problem*)):ti,ab,kw	2 148
	#45	'internet addiction'/de	2 944
	#46	(cyberaddiction OR 'cyber addiction'):ti,ab,kw	41
	#47	((constant OR hyper OR permanent) NEAR/3 connectivity):ti,ab,kw	326
	#48	'game addiction'/de	1 471
	#49	'pathological gambling'/de	6 540
	#50	((digital OR electronic OR internet OR online) NEAR/3 (abus* OR addict* OR bet OR bets OR betting OR betted OR bettor OR bettors OR bing* OR dependenc* OR overus* OR misus* OR disorder* OR compuls* OR obsessi* OR excess* OR pathologic* OR problem*)):ti,ab,kw	8 053
	#51	'mobile phone addiction'/de	457
	#52	((mobile OR smartphone* OR chat) NEAR/3 (abus* OR addict* OR dependenc* OR overus* OR compuls* OR obsessi* OR excess* OR patholog* OR disorder* OR problem*)):ti,ab,kw	1570
	#53	'social media addiction'/de	190
	#54	(('social media' OR 'social network*') NEAR/3 (abus* OR addict* OR dependenc* OR excess* OR overus* OR compuls* OR obsessi* OR problem* OR disorder*)):ti,ab,kw	837
	#55	wager*:ti,ab,kw	408
**Dependences combined**	#56	#27 OR #28 OR #29 OR #30 OR #31 OR #32 OR #33 OR #34 OR #35 OR #36 OR #37 OR #38 OR #39 OR #40 OR #41 OR #42 OR #43 OR #44 OR #45 OR #46 OR #47 OR #48 OR #49 OR #50 OR #51 OR #52 OR #53 OR #54 OR #55	718 065
3rd Treatments	#57	psychotherap*:ti,ab,kw	70 041
	#58	'psychotherapy'/de	101 742
	#59	'short term psychotherapy'/de	350
	#60	therap*:ti,ab,kw	4 511 809

	#61	'therapy'/de	1 281 313
	#62	'rational emotive behavior therapy'/de	184
	#63	'cognitive behavioral therapy'/de	16 474
	#64	'dialectical behavior therapy'/de	811
	#65	'cognitive therapy'/de	43 685
	#66	'acceptance and commitment therapy'/de	1 758
	#67	'group therapy'/de	22 995
	#68	intervention*:ti,ab,kw	1 564 847
	#69	'intervention'/de	91
	#70	'psychosocial intervention'/de	479
	#71	'mental health care'/de	30 372
	#72	'mental health care':ti,ab,kw	15 932
	#73	'mindfulness'/de	10 482
	#74	mindfulness:ti,ab,kw	12 427
	#75	(program OR programs* OR programme OR programmes):ti,ab,kw	1 137 059
	#76	'treatment'/de	5 836
	#77	treatment*:ti,ab,kw	6 900 206
Treatments combined	#78	#57 OR #58 OR #59 OR #60 OR #61 OR #62 OR #63 OR #64 OR #65 OR #66 OR #67 OR #68 OR #69 OR #70 OR #71 OR #72 OR #73 OR #74 OR #75 OR #76 OR #77	11 428 469
all concepts **combined**	#79	#26 AND #56 AND #78	3 305
	#80	#79 AND (2000:py OR 2001:py OR 2002:py OR 2003:py OR 2004:py OR 2005:py OR 2006:py OR 2007:py OR 2008:py OR 2009:py OR 2010:py OR 2011:py OR 2012:py OR 2013:py OR 2014:py OR 2015:py OR 2016:py OR 2017:py OR 2018:py OR 2019:py OR 2020:py OR 2021:py OR 2022:py)	3 053
**Limited**	#81	#70 AND [embase]/lim	2655

PROQUEST DISSERTATIONS & THESES GLOBAL

Database : no limit
Period of coverage:1939-2022
Update frequency: Monthly
Date: January 31, 2022

Concepts	#	Query	Results

			(on January 31, 2022)
1st Emotion regulation	S1	AB,TI(affect* NEAR/3 control*)	4 599
	S2	AB,TI(affect* NEAR/3 regulation*)	2 430
	S3	AB,TI(affect* NEAR/3 response*)	8 252
	S4	AB,TI(alexithymia)	659
	S5	AB,TI(emotion* NEAR/3 adjust*)	1 542
	S6	AB,TI("emotion* awareness")	390
	S7	AB,TI(emotion* NEAR/3 control*)	2 431
	S8	AB,TI(emotion* NEAR/3 disturbance*)	1 263
	S9	AB,TI(emotion* NEAR/3 dysregulation*)	729
	S10	AB,TI(emotion* NEAR/3 instabilit*)	184
	S11	AB,TI("emotion* manag*t")	369
	S12	AB,TI(emotion* NEAR/3 reactivit*)	751
	S13	AB,TI(emotion* NEAR/3 recognition)	1 246
	S14	AB,TI(emotion* NEAR/3 regulation)	4 394
	S15	AB,TI("emotion* response*")	3 504
	S16	AB,TI(emotion* NEAR/3 stabilit*)	811
	S17	AB,TI(emotion* NEAR/3 state*)	3 618
Emotion regulation combined	S18	1 OR 2 OR 3 OR 4 OR 5 OR 6 OR 7 OR 8 OR 9 OR 10 OR 11 OR 12 OR 13 OR 14 OR 15 OR 16 OR 17	33 146
2nd Dependences	S19	AB,TI(alcohol NEAR/3 (binge OR binges OR binged OR bingeing OR abuse OR abused OR abuses OR abusing OR misuse OR misusing OR misusage OR misused OR addict OR addicts OR addicted OR addictive OR addicting OR addiction OR addictions OR dependence OR dependency OR dependencies OR disorder OR disorders OR disordered OR problem OR problems OR problematic OR problematical))	7 376
	S20	AB,TI(alcoholic OR alcoholism)	8 266
	S21	AB,TI(gambl* NEAR/3 (compulsive OR obsessive OR excessive OR pathologic OR pathological OR pathologically OR addict OR addicts OR addicted OR addictive OR addicting OR addiction OR addictions OR dependence OR dependency OR dependencies OR disorder OR disorders OR	670

		disordered OR problem OR problems OR problematic OR problematical))	
	S22	AB,TI(computer* NEAR/3 (compulsive OR obsessive OR excessive OR overuse OR overuses OR overusing OR overused OR abuse OR abused OR abuses OR abusing OR misuse OR misusing OR misusage OR misused OR addict OR addicts OR addicted OR addictive OR addicting OR addiction OR addictions OR dependence OR dependency OR dependencies OR disorder OR disorders OR disordered OR problem OR problems OR problematic OR problematical))	3 963
	S23	AB,TI(cyberaddiction OR "cyber addiction")	2
	S24	AB,TI((constant OR hyper OR permanent) NEAR/3 connectivity)	77
	S25	AB,TI((digital OR electronic OR internet OR online) NEAR/3 (bet OR bets OR betting OR betted OR bettor OR bettors OR pathologic OR pathological OR pathologically OR compulsive OR obsessive OR excessive OR overuse OR overuses OR overusing OR overused OR abuse OR abused OR abuses OR abusing OR misuse OR misusing OR misusage OR misused OR addict OR addicts OR addicted OR addictive OR addicting OR addiction OR addictions OR dependence OR dependency OR dependencies OR disorder OR disorders OR disordered OR problem OR problems OR problematic OR problematical))	4 538
	S26	AB,TI(drug* NEAR/3 (use OR used OR uses OR using OR usage OR abuse OR abused OR abuses OR abusing OR misuse OR misusing OR misusage OR misused OR addict OR addicts OR addicted OR addictive OR addicting OR addiction OR addictions OR dependence OR dependency OR dependencies OR disorder OR disorders OR disordered OR problem OR problems OR problematic OR problematical))	24 903
	S27	AB,TI((drink* OR drank OR drunk) NEAR/3 (binge OR binges OR binged OR bingeing OR abuse OR abused OR abuses OR abusing OR misuse OR misusing OR misusage OR misused OR addict OR addicts OR addicted OR addictive OR	2 364

		addicting OR addiction OR addictions OR dependence OR dependency OR dependencies OR disorder OR disorders OR disordered OR problem OR problems OR problematic OR problematical))	
	S28	AB,TI((mobile OR smartphone* OR chat) NEAR/3 (compulsive OR obsessive OR excessive OR overuse OR overuses OR overusing OR overused OR abuse OR abused OR abuses OR abusing OR misuse OR misusing OR misusage OR misused OR addict OR addicts OR addicted OR addictive OR addicting OR addiction OR addictions OR dependence OR dependency OR dependencies OR disorder OR disorders OR disordered OR problem OR problems OR problematic OR problematical))	1 051
	S29	AB,TI(("social media" OR "social network*") NEAR/3 (compulsive OR obsessive OR excessive OR overuse OR overuses OR overusing OR overused OR abuse OR abused OR abuses OR abusing OR addict OR addicts OR addicted OR addictive OR addicting OR addiction OR addictions OR dependence OR dependency OR dependencies OR disorder OR disorders OR disordered OR problem OR problems OR problematic OR problematical))	463
	S30	AB,TI(substance* NEAR/3 (use OR used OR uses OR using OR usage OR abuse OR abused OR abuses OR abusing OR misuse OR misusing OR misusage OR misused OR addict OR addicts OR addicted OR addictive OR addicting OR addiction OR addictions OR dependence OR dependency OR dependencies OR disorder OR disorders OR disordered OR problem OR problems OR problematic OR problematical))	14 963
	S31	AB,TI(toxicoman*)	119
	S32	AB,TI(wager OR wagers OR wagered OR wagering)	298
Dependences combined	S33	19 OR 20 OR 21 OR 22 OR 23 OR 24 OR 25 OR 26 OR 27 OR 28 OR 29 OR 30 OR 31 OR 32	58 426
**3rd** Treatments	S34	AB,TI(psychotherap*)	13 697
	S35	AB,TI(therap*)	149 498
	S36	AB,TI(intervention*)	164 304

	S37	AB,TI("mental health care")	2 030
	S38	AB,TI(mindfulness)	4 698
	S39	AB,TI(program OR programs* OR programme OR programmes)	408 599
	S40	AB,TI(treatment*)	352 928
Treatments combined	S41	34 OR 35 OR 36 OR 37 OR 38 OR 39 OR 40	897 315
**FINAL SEARCH** all concepts **combined**	S42	18 AND 33 AND 41	591
	S43	18 AND 33 AND 41<br>Restriction : 2000-01-01 - 2022-01-31	460

PSYCHOLOGY AND BEHAVIORAL SCIENCES COLLECTION

Database : no limit
Period of coverage: 1930-2022
Update frequency: daily
Date: January 31, 2022

Concepts	#	Query	Results (on January 31, 2022)
1st Emotion regulation	S1	AB (affect* N3 control*) OR TI (affect* N3 control*)	1 270
	S2	AB (affect* N3 regulation*) OR TI (affect* N3 regulation*)	833
	S3	AB (affect* N3 response*) OR TI (affect* N3 response*)	2 091
	S4	AB (alexithymia) OR TI (alexithymia)	630
	S5	AB (emotion*) OR TI (emotion*)	52 545
	S6	AB (emotion* N3 adjust*) OR TI (emotion* N3 adjust*)	689
	S7	AB ("emotion* awareness") OR TI ("emotion* awareness")	226
	S8	AB (emotion* N3 control*) OR TI (emotion* N3 control*)	1 523
	S9	AB (emotion* N3 disturbance*) OR TI (emotion* N3 disturbance*)	771
	S10	AB (emotion* N3 dysregulation*) OR TI (emotion* N3 dysregulation*)	762
	S11	AB (emotion* N3 instabilit*) OR TI (emotion* N3 instabilit*)	130
	S12	AB ("emotion* manag*t") OR TI ("emotion* manag*t")	109
	S13	AB (emotion* N3 reactivit*) OR TI (emotion* N3 reactivit*)	636

	S14	AB (emotion* N3 recognition) OR TI (emotion* N3 recognition)	1 021
	S15	AB (emotion* N3 regulation) OR TI (emotion* N3 regulation)	3 662
	S16	AB ("emotion* response*") OR TI ("emotion* response*")	1 416
	S17	AB (emotion* N3 stabilit*) OR TI (emotion* N3 stabilit*)	457
	S18	AB (emotion* N3 state*) OR TI (emotion* N3 state*)	1 758
	S19	(ZU "alexithymia")	782
	S20	(ZU "emotion recognition")	210
	S21	(ZU "emotional shutdown (psychology)")	1
	S22	(ZU "emotional stability")	65
	S23	(ZU "emotional state")	262
Emotion regulation combined	S24	S1 OR S2 OR S3 OR S4 OR S5 OR S6 OR S7 OR S8 OR S9 OR S10 OR S11 OR S12 OR S13 OR S14 OR S15 OR S16 OR S17 OR S18 OR S19 OR S20 OR S21 OR S22 OR S23	56 169
**2nd** Dependences	S25	AB (alcohol N3 (misus* OR abus* OR addict* OR dependenc* OR disorder* OR bing* OR problem*)) OR TI (alcohol N3 (misus* OR abus* OR addict* OR dependenc* OR disorder* OR bing* OR problem*))	9 479
	S26	AB (alcoholic OR alcoholism) OR TI (alcoholic OR alcoholism)	4 935
	S27	AB (gambl* N3 (addict* OR compulsive OR obsessi* OR dependenc* OR disorder* OR excessi* OR problem* OR pathologic*)) OR TI (gambl* N3 (addict* OR compulsive OR obsessi* OR dependenc* OR disorder* OR excessi* OR problem* OR pathologic*))	941
	S28	AB (computer* N3 (misus* OR abus* OR addict* OR dependenc* OR disorder* OR compuls* OR obsessi* OR excessive OR overus* OR problem*)) OR TI (computer* N3 (misus* OR abus* OR addict* OR dependenc* OR disorder* OR compuls* OR obsessi* OR excessive OR overus* OR problem*))	417
	S29	AB (cyberaddiction OR "cyber addiction") OR TI (cyberaddiction OR "cyber addiction")	0
	S30	AB ((constant OR hyper OR permanent) N3 connectivity) OR TI ((constant OR hyper OR permanent) N3 connectivity)	16
	S31	AB ((digital OR electronic OR internet OR online) N3 (abus* OR addict* OR bet OR bets OR betting OR betted OR bettor OR bettors OR bing* OR dependenc* OR overus* OR misus* OR disorder* OR compuls* OR	1 334

		obsessi* OR excess* OR pathologic* OR problem*)) OR TI ((digital OR electronic OR internet OR online) N3 (abus* OR addict* OR bet OR bets OR betting OR betted OR bettor OR bettors OR bing* OR dependenc* OR overus* OR misus* OR disorder* OR compuls* OR obsessi* OR excess* OR pathologic* OR problem*))	
	S32	AB (drug* N3 (use* OR usage OR misuse* OR abus* OR addict* OR depedenc* OR disorder* OR problem*)) OR TI (drug* N3 (use* OR usage OR misuse* OR abus* OR addict* OR depedenc* OR disorder* OR problem*))	19 864
	S33	AB ((drink* OR drank OR drunk) N3 (bing* OR misus* OR abus* OR addict* OR dependenc* OR disorder* OR problem*)) OR TI ((drink* OR drank OR drunk) N3 (bing* OR misus* OR abus* OR addict* OR dependenc* OR disorder* OR problem*))	2 686
	S34	AB ((mobile OR smartphone* OR chat) N3 (abus* OR addict* OR dependenc* OR overus* OR compuls* OR obsessi* OR excess* OR patholog* OR disorder* OR problem*)) OR TI ((mobile OR smartphone* OR chat) N3 (abus* OR addict* OR dependenc* OR overus* OR compuls* OR obsessi* OR excess* OR patholog* OR disorder* OR problem*))	175
	S35	AB (("social media" OR "social network*") N3 (abus* OR addict* OR dependenc* OR overus* OR compuls* OR obsessi* OR excess* OR problem* OR disorder*)) OR TI (("social media" OR "social network*") N3 (abus* OR addict* OR dependenc* OR excess* OR overus* OR compuls* OR obsessi* OR problem* OR disorder*))	147
	S36	AB (substance* N3 (use* OR usage OR misuse* OR abus* OR addict* OR dependenc* OR disorder* OR problem*)) OR TI (substance* N3 (use* OR usage OR misuse* OR abus* OR addict* OR dependenc* OR disorder* OR problem*))	15 920
	S37	AB (toxicoman*) OR TI (toxicoman*)	181
	S38	AB (wager*) OR TI (wager*)	110
	S39	(ZU "alcohol drinking")	7 356
	S40	(ZU "designer drugs")	47
	S41	(ZU "drug abuse")	5 317
	S42	(ZU "gambler psychology")	18
	S43	(ZU "gamblers")	114

	S44	(ZU "gambling")	807
	S45	(ZU "gambling behavior")	121
	S46	(ZU "gambling -- psychological aspects")	174
	S47	(ZU "impulse control disorders")	342
	S48	(ZU "internet addiction")	478
	S49	(ZU "internet addicts")	55
	S50	(ZU "internet gambling")	67
	S51	(ZU "internet games")	0
	S52	(ZU "internet pornography addiction")	69
	S53	(ZU "internet users")	524
	S54	(ZU "online social networks")	588
	S55	(ZU "video lottery")	1
	S56	(ZU "video lottery terminals")	6
	S57	(ZU "video poker")	3
	S58	(ZU "video slot machines")	1
	S59	(ZU "video gamers")	121
	S60	(ZU "video games")	1 177
	S61	(ZU "video games -- psychological aspects")	151
Dependences combined	S62	S25 OR S26 OR S27 OR S28 OR S29 OR S30 OR S31 OR S32 OR S33 OR S34 OR S35 OR S36 OR S37 OR S38 OR S39 OR S40 OR S41 OR S42 OR S43 OR S44 OR S45 OR S46 OR S47 OR S48 OR S49 OR S50 OR S51 OR S52 OR S53 OR S54 OR S55 OR S56 OR S57 OR S58 OR S59 OR S60 OR S61	51 712
3rd Treatments	S63	AB (psychotherap*) OR TI (psychotherap*)	18 762
	S64	AB (therap*) OR TI (therap*)	81 304
	S65	AB (intervention*) OR TI (intervention*)	83 156
	S66	AB ("mental health care") OR TI ("mental health care")	3 122
	S67	AB (mindfulness) OR TI (mindfulness)	2 438
	S68	AB (program OR programs* OR programme OR programmes) OR TI (program OR programs* OR programme OR programmes)	79 610
	S69	AB (treatment*) OR TI (treatment*)	130 374
	S70	(ZU "brief psychotherapy")	319
	S71	(ZU "cognitive therapy")	6 596
	S72	(ZU "cognitive-experiential psychotherapy")	15
	S73	(ZU "dialectical behavior therapy")	232

	S74	(ZU "group psychotherapy")	2 150
	S75	(ZU "interventional therapies llc")	1
	S76	(ZU "mindfulness")	1 472
	S77	(ZU "mindfulness-based cognitive therapy")	249
	S78	(ZU "psychotherapy")	15 686
	S79	(ZU "rational emotive behavior therapy")	139
	S80	(ZU "treatment programs")	906
Treatments combined	S81	S63 OR S64 OR S65 OR S66 OR S67 OR S68 OR S69 OR S70 OR S71 OR S72 OR S73 OR S74 OR S75 OR S76 OR S77 OR S78 OR S79 OR S80	300 897
**FINAL SEARCH** all concepts **combined**	S82	S24 AND S62 AND S81	1093
	S83	S24 AND S80 AND S81<br><br>Opérateurs de restriction - Date de publication: 20000101-20221231	1 004

WEB OF SCIENCE (Core collection)

Database : limited to
Science Citation Index Expanded (SCI-EXPANDED) Period of coverage: 1900-present
Social Sciences Citation Index (SSCI) Period of coverage: 1900-present
Emerging Sources Citation Index (ESCI) Period of coverage: 2005-present

Update frequency: daily
Date: January 31, 2022

**Concepts**	**#**	**Query (TOPIC)**	**Results (on January 31, 2022)**
1st Emotion regulation	1.	TS=(affect* NEAR/3 control*)	22 959
	2.	TS= (affect* NEAR/3 regulation*)	11 126
	3.	TS= (affect* NEAR/3 response*)	47 592
	4.	TS=alexithymia	6 336
	5.	TS= (emotion* NEAR/3 adjust*)	2 948
	6.	TS="emotion* awareness"	1 298
	7.	TS=(emotion* NEAR/3 control*)	9 197
	8.	TS=(emotion* NEAR/3 disturbance*)	3 499
	9.	TS=(emotion* NEAR/3 dysregulation*)	4 262
	10.	TS=(emotion* NEAR/3 instabilit*)	927

	11.	TS="emotion* manag* "	1 198
	12.	TS=(emotion* NEAR/3 reactivit*)	4 208
	13.	TS=(emotion* NEAR/3 recognition)	11 154
	14.	TS=(emotion* NEAR/3 regulation)	26 544
	15.	TS="emotion* response*"	11 292
	16.	TS=(emotion* NEAR/3 stabilit*)	2 670
	17.	TS=(emotion* NEAR/3 state*)	14 587
Emotion regulation combined	18.	#1 OR #2 OR #3 OR #4 OR #5 OR #6 OR #7 OR #8 OR #9 OR #10 OR #11 OR #12 OR #13 OR #14 OR #15 OR #16 OR #17	163 520
**2nd** Dependences	19.	TS=(alcohol NEAR/3 (misus* OR abus* OR addict* OR dependenc* OR disorder* OR bing* OR problem*))	73 155
	20.	TS=((drink* OR drank OR drunk) NEAR/3 (bing* OR misus* OR abus* OR addict* OR dependenc* OR disorder* OR problem*))	19 864
	21.	TS=(alcoholic OR alcoholism)	114 577
	22.	TS=(drug* NEAR/3 (use* OR usage OR misuse* OR abus* OR addict* OR depedenc* OR disorder* OR problem*))	290 316
	23.	TS=(substance* NEAR/3 (use* OR usage OR misuse* OR abus* OR addict* OR dependenc* OR disorder* OR problem*))	130 203
	24.	TS=toxicoman*	193
	25.	TS=(gambl* NEAR/3 (addict* OR compulsive OR obsessi* OR dependenc* OR disorder* OR excessi* OR problem* OR pathologic*))	7 509
	26.	TS=(computer* NEAR/3 (misus* OR abus* OR addict* OR dependenc* OR disorder* OR compuls* OR obsessi* OR excessive OR overus* OR problem*))	8 444
	27.	TS=(cyberaddiction OR "cyber addiction")	34
	28.	TS=((constant OR hyper OR permanent) NEAR/3 connectivity)	561
	29.	TS=((digital OR electronic OR internet OR online) NEAR/3 (abus* OR addict* OR bet OR bets OR betting OR betted OR bettor OR bettors OR bing* OR dependenc* OR overus* OR misus* OR disorder* OR compuls* OR obsessi* OR excess* OR pathologic* OR problem*))	24 204
	30.	TS=((mobile OR smartphone* OR chat) NEAR/3 (abus* OR addict* OR dependenc* OR overus* OR compuls* OR obsessi* OR excess* OR patholog* OR disorder* OR problem*))	5 364

	31.	TS=(("social media" OR "social network*") NEAR/3 (abus* OR addict* OR dependenc* OR excess* OR overus* OR compuls* OR obsessi* OR problem* OR disorder*))	2 241
	32.	TS=wager*	1 105
Dependences combined	33.	#19 OR #20 OR #21 OR #22 OR #23 OR #24 OR #25 OR #26 OR #27 OR #28 OR #29 OR #30 OR #31 OR #32	574 787
**3**[rd] Treatments	34.	TS=psychotherap*	87 939
	35.	TS=therap*	3 499 916
	36.	TS=intervention*	1 390 274
	37.	TS="mental health care"	16 143
	38.	TS=mindfulness	21 250
	39.	TS=(program OR programs* OR programme OR programmes)	1 807 935
	40.	TS=treatment*	5 521 601
Treatments combined	41.	#34 OR #35 OR #36 OR #37 OR #38 OR #39 OR #40	9 789 641
**FINAL SEARCH** all concepts **combined**	42.	#18 AND #33 AND #41	3 183
	43.	#42 AND Timespan: 2000-01-01 to 2022-01-31 (Publication Date)	3 019
